# Recent Advances in Carbon Quantum Dot-Enhanced Stimuli-Sensitive Hydrogels: Synthesis, Properties, and Applications

**DOI:** 10.3390/gels12040332

**Published:** 2026-04-16

**Authors:** Mingna Li, Yanlin Du, Yunfeng He, Jiahua He, Du Ji, Qing Sun, Yongshuai Ma, Linyan Zhou, Yongli Jiang, Junjie Yi

**Affiliations:** 1 Faculty of Food Science and Engineering, Kunming University of Science and Technology, Kunming 650500, China; limingna1102@163.com (M.L.); duyanlin_work@163.com (Y.D.); mays@kust.edu.cn (Y.M.); 20180012@kust.edu.cn (L.Z.); junjieyi@kust.edu.cn (J.Y.); 2 Yunnan Key Laboratory of Plateau Food Advanced Manufacturing, Kunming 650500, China; 3 Institute of Plateau Biology of Diqing Tibetan Autonomous Prefecture, Shangri La 674499, China; 4 School of Food and Biological Engineering, Jiangsu University, Zhenjiang 212013, China; qing.sun@ujs.edu.cn

**Keywords:** carbon quantum dots, stimuli-sensitive hydrogels, smart materials, drug delivery, biosensing

## Abstract

Carbon quantum dots (CQDs) and stimuli-responsive hydrogels are advanced functional materials whose hybridization yields CQD-enhanced stimuli-sensitive hydrogels, opening new interdisciplinary avenues for smart material applications. This review systematically summarizes the latest advances in these composites, focusing on synthetic strategies, structure–property modulation mechanisms, and practical applications. Distinct from existing reviews that either investigate CQDs or hydrogels independently or discuss their composites in a single research field, this work features core novelties in integration strategy, application scope and critical analysis: it systematically compares the advantages, limitations and applicable scenarios of three typical CQD–hydrogel integration approaches (physical entrapment, in situ synthesis, covalent conjugation), comprehensively covers the multi-field application progress of the composites and conducts in-depth cross-field analysis of their common scientific issues and technical bottlenecks. By incorporating CQDs, the composites achieve remarkable performance optimizations: 40% improved mechanical toughness, sub-ppm-level heavy metal-sensing sensitivity, and over 80% organic dye photocatalytic degradation efficiency, addressing pure hydrogels’ inherent limitations of insufficient strength and single functionality. These enhancements enable sophisticated applications in biomedical field (real-time biosensing, controlled drug delivery), environmental remediation (pollutant detection/degradation), energy storage, and flexible electronics. The synergistic interplay between CQDs and hydrogels facilitates precise single/multi-stimulus responsiveness (pH, temperature, light), a pivotal advance for precision medicine and intelligent environmental monitoring. Despite promising progress, the large-scale practical application of CQD–hydrogel composites still faces prominent challenges: the difficulty in scalable fabrication with the uniform dispersion of CQDs in hydrogel matrices, poor long-term stability of most composites under physiological cyclic stress (service life < 6 months in practical tests), and low accuracy in discriminating multi-stimuli in complex real-world matrices. Future research should prioritize biomass-based eco-friendly CQD synthesis, machine learning-aided multimodal responsive systems, and 3D bioprinting for scalable manufacturing.

## 1. Introduction

Stimuli-responsive hydrogels show substantial potential in biomedical and environmental engineering, including targeted drug delivery, tissue engineering, and smart sensing [1,2], but their potential is limited by their inadequate mechanical strength under cyclic stress and insufficient functional versatility beyond basic stimulus–response behaviors. To address these constraints, carbon quantum dots (CQDs)—zero-dimensional carbon nanomaterials (typically 2–8 nm in diameter) with unique photoluminescence, tunable surface chemistry, and exceptional electron mobility [3,4]—have been integrated as multifunctional nano-modifiers. Synthesizable via top-down (e.g., laser ablation of graphite) or bottom-up (e.g., hydrothermal carbonization of organic precursors) approaches, CQDs interact with polymer chains through surface functional groups (e.g., –COOH, –OH) to reinforce hydrogel networks and introduce optoelectronic responsivity and photocatalytic functionality [5,6], expanding the resulting nanocomposites into advanced domains such as real-time biosensing and photocatalytic wastewater treatment [7,8].

Although CQDs and hydrogels have been extensively studied individually, their synergistic integration into stimuli-responsive hydrogels remains an underexplored frontier [9]. Existing reviews suffer from three critical deficiencies: first, they fail to systematically compare different CQD–hydrogel integration strategies, leading to the unclear understanding of performance trade-offs and applicable scenarios; second, they lack in-depth elaboration on the molecular-level “structure–property–performance” correlation of composites, hindering rational material design; third, most focus on single-field applications without cross-field critical analysis of common scientific problems and technical bottlenecks [10,11]. This leaves a prominent research gap: the lack of a unified interdisciplinary framework that consolidates the diverse fabrication strategies, coordination mechanisms, and multi-field applications of CQD–hydrogel composites.

**Figure 1:**
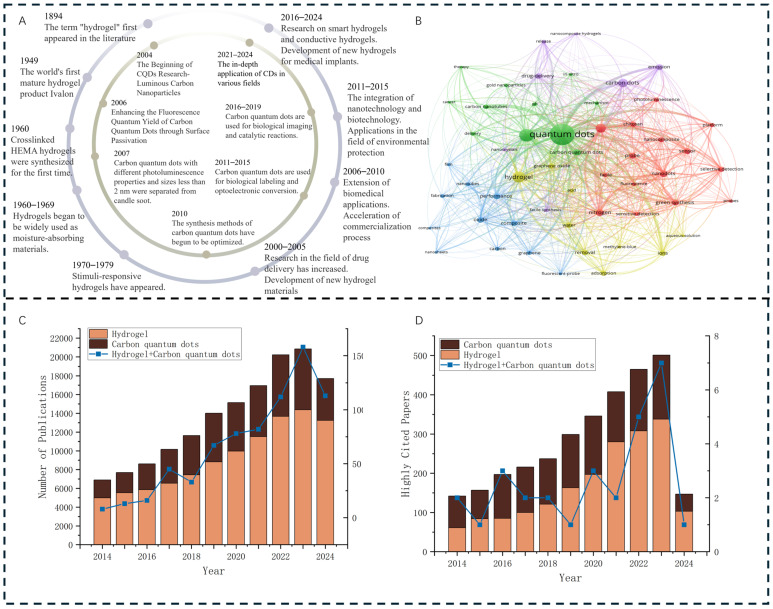
(**A**) The development history of carbon quantum dots and hydrogels. (**B**) Correlation map of keywords related to “carbon quantum dots” and “hydrogels” in research papers (retrieved from www.webofscience.com). (**C**) Total number of publications and (**D**) highly cited articles related to hydrogels and carbon quantum dots in the last 10 years. According to Web of Science search on 11 June 2025.

The core objective of this review is to systematically address the aforementioned research gaps and provide a comprehensive, in-depth academic reference for the subsequent research, performance regulation, and practical application of CQD–hydrogel composites. Its scope is clearly defined as follows: (1) summarize the synthesis methods of CQDs and their key physicochemical characteristics essential for hydrogel integration; (2) classify the main types of stimuli-responsive hydrogels and elaborate their core stimulus–response mechanisms; (3) analyze typical fabrication techniques for CQD–hydrogel integration and the corresponding structure–property regulation rules; (4) critically review the latest research progress of CQD–hydrogel composites in four major fields (biomedicine, environmental remediation, energy storage, smart materials); and (5) identify the current core limitations and practical challenges of the composite system, and outline feasible future research directions emphasizing multi-stimuli responsiveness, scalable green synthesis, and advanced manufacturing approaches (Figure 1). By achieving these objectives, this review seeks to bridge the gap between fundamental research and practical applications, offering valuable insights for researchers, engineers, and industry professionals in materials science, nanotechnology, and biomedical engineering [12].

## 2. Synthesis of Carbon Quantum Dot-Enhanced Stimuli-Sensitive Hydrogels

### 2.1. Synthesis and Characteristics of CQDs

#### 2.1.1. Top-Down Synthesis Methods

Top-down synthesis fragments bulk carbon materials (e.g., graphite) into CQDs through physical or chemical exfoliation techniques. Among these, arc discharge vaporizes carbon sources via high-temperature electric arcs, enabling scalable production but requiring extensive post-synthesis purification [13,14]. Laser ablation, pioneered by Sun et al. (2006), utilizes high-energy lasers to irradiate carbon targets, achieving precise control over CQDs’ size and morphology, albeit with higher operational costs [15]. Electrochemical synthesis oxidizes carbon precursors in aqueous electrolytes, offering simplicity in setup yet limited production yields [16]. Additionally, chemical oxidation selectively etches carbon precursors through strong oxidizing agents (e.g., HNO_3_), yielding hydrophilic CQDs with tunable surface groups [17].

A critical comparison of these methods reveals clear trade-offs: arc discharge prioritizes scalability over purity (65–90% purity across the literature), laser ablation emphasizes precision over cost-efficiency, electrochemical synthesis balances simplicity with low yield, and chemical oxidation offers functional tunability at the expense of environmental impact. Notably, the structural features tailored by each approach directly influence compatibility with hydrogel networks—laser-ablated CQDs with a narrow size distribution (<5 nm) form uniform crosslinking site, while chemically oxidized CQDs with abundant carboxyl groups (-COOH) strengthen hydrogen bonding with hydroxyl-rich hydrogels (e.g., chitosan, alginate), amplifying mechanical performance through interfacial synergy. Chemical ablation methods, such as the conversion of petroleum coke into N-doped CQDs (N-CQDs), further demonstrate versatility by incorporating heteroatoms during fragmentation [18]. Recent advances also highlight intercalation-driven approaches, where layered carbon materials are expanded and exfoliated using intercalants like H_2_SO_4_/KMnO_4_, resulting in size-controlled CQDs [19]. Sustainable routes, such as the synthesis of N-doped micropore CQDs (NM-CQDs) from waste biomass, align with green chemistry principles [20]. Discrepancies in reported performance—Wang et al. (2025) achieved 90% purity via arc discharge with optimized post-treatment [21], while Li et al. (2022) reported only 65% purity without purification optimization—underscore the need for standardized protocols to ensure consistent integration with hydrogels [22].

#### 2.1.2. Bottom-Up Synthesis Methods

Bottom-up strategies assemble molecular or polymeric carbon precursors (e.g., citric acid, biomass) into CQDs via controlled carbonization and passivation. Hydrothermal synthesis, exemplified by the conversion of carrot-derived precursors into CQDs, dissolves precursors under high temperature and pressure, producing uniform nanoparticles under mild conditions, albeit with prolonged reaction times [23]. Microwave-assisted methods utilize rapid electromagnetic heating to accelerate reaction kinetics, achieving high yields within minutes but requiring specialized equipment [24]. For instance, microwave treatment of lemon juice and ammonia generates nitrogen-doped CQDs with enhanced fluorescence [25]. Sonochemical approaches, such as the hydrothermal synthesis of CQDs from corn cob biomass, leverage ultrasonic waves to enhance precursor decomposition and nucleation, improving crystallinity [26]. Biomass-derived routes, including broccoli-based CQDs, emphasize sustainability by repurposing natural waste into functional nanomaterials [27]. Pyrolysis thermally decomposes precursors in inert atmospheres, offering cost-effective synthesis but broader size distributions.

Heteroatom doping (e.g., nitrogen or sulfur) modifies CQDs’ electronic structure, a key factor in their performance within hydrogels—N-doped CQDs exhibit 2–3 fold higher electron transfer efficiency than undoped counterparts, translating to enhanced photocatalytic activity in composite systems. This is evident in metal–organic framework (MOF)-templated CQDs, which exhibit tunable photoluminescence and high quantum yields (>70%) but rely on expensive precursors [28]. Compared to top-down methods, bottom-up strategies generally offer higher purity (>85%) and functional uniformity but face greater scalability barriers, with precursor composition and reaction parameters directly governing compatibility with hydrogel matrices.

#### 2.1.3. Physicochemical Properties and Applications

**Figure 2:**
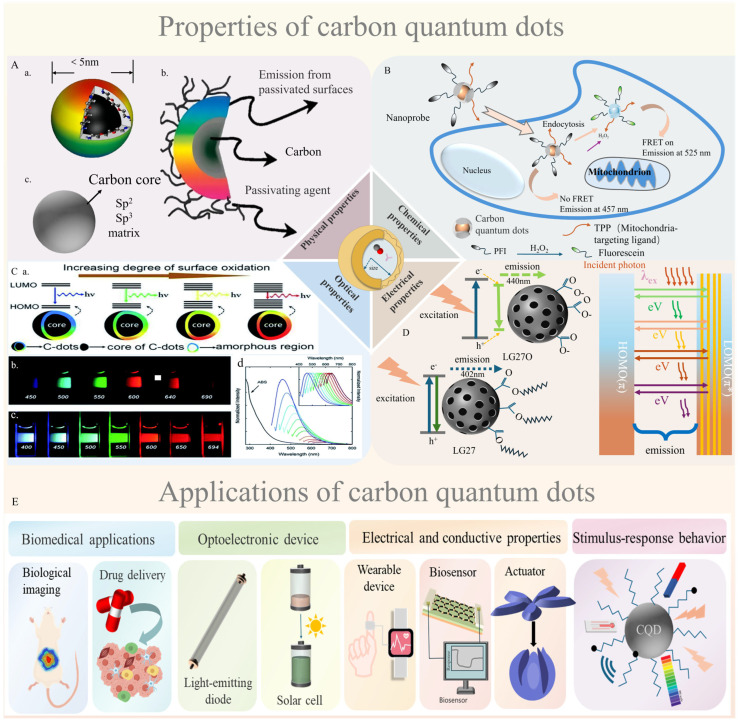
(**A**) Schematic illustrations of CQDs’ structural configurations: (**a**) ethylenediamine (EDA)-functionalized CQDs with a “core–shell” structure—an amorphous carbon core tethered to EDA molecules, enabling pH-responsive charge transfer via protonation/deprotonation of amino groups; (**b**) classic CQD structure with sp^2^/sp^3^-hybridized carbon networks and oxygen-containing surface moieties (–COOH, –OH), facilitating non-covalent interactions with hydrogel polymers; (**c**) crystalline carbon core of CQDs, whose degree of graphitization modulates electron transfer efficiency and photoluminescence (PL) stability. (**B**) Biocompatibility and drug delivery mechanism of CQDs: CQDs’ high surface area (100–500 m^2^/g) and abundant functional groups enable efficient drug loading (e.g., doxorubicin) via electrostatic adsorption or π-π stacking, while their low cytotoxicity (IC_50_ > 100 μg/mL) supports in vivo applications. (**C**) Tunable PL properties of CQDs: (**a**) mechanism for oxidation-dependent PL modulation—controlled oxidation introduces surface defects that red-shift emission wavelengths by regulating electron–hole recombination pathways; (**b**) aqueous solution of PEG1500N-attached CQDs (λex = 400 nm) exhibits excitation-dependent emission across visible wavelengths, supporting multiplexed sensing; (**c**) photographs of CQDs solutions under specified excitation wavelengths, demonstrating naked-eye-distinguishable fluorescence; (**d**) absorbance (ABS) and PL emission spectra of propionylethyleneimine-co-ethyleneimine CQDs, with emission peaks shifting progressively with excitation wavelength (400–500 nm in 20 nm increments). (**D**) PL mechanism of LG27 (pristine CQDs) and LG27O (NaOH-treated CQDs): NaOH-induced surface carboxylation increases electron-donating groups, suppressing non-radiative recombination and enhancing quantum yield by 20–30%. (**E**) Versatile applications of CQDs in hydrogel composites, including bioimaging, drug delivery, environmental sensing, and photocatalysis—each enabled by CQDs’ tunable optical, electrical, or chemical properties.

CQDs are zero-dimensional nanomaterials (<10 nm) characterized by high surface-to-volume ratios and quantum confinement effects, which govern their size- and surface-dependent optical/electronic behaviors [15,29]. Structurally, CQDs typically exhibit a “core–shell” configuration, where a crystalline carbon core (Figure 2A(c)) is surrounded by functionalized surface groups. For instance, ethylenediamine (EDA)-functionalized CQDs (Figure 2A(a)) feature an amorphous carbon core with EDA molecules tethered to the surface, enabling pH-responsive charge transfer, while early CQD models (Figure 2A(b)) emphasized sp^2^/sp^3^-hybridized carbon networks with oxygen-containing surface moieties [30,31]. The optical properties of CQDs include tunable photoluminescence (PL) spanning visible to near-infrared regions, driven by π-π* and n-π* electronic transitions [32]. PL modulation can be achieved through surface oxidation states, as demonstrated by Ding et al. (2016) (Figure 2C(a)), where controlled oxidation enhances emission red-shifting via defect state engineering [33]. Additionally, excitation-dependent PL behavior (Figure 2C(b,c)) and up-conversion luminescence (Figure 2C(d)) enable multiplexed sensing and bioimaging [15]. Surface functionalization (e.g., PEG1500N grafting in Figure 2C(b)) further stabilizes CQDs in aqueous media while preserving luminescence. Notably, NaOH treatment of LG27 CQDs (Figure 2D) induces surface carboxylation, altering electron–hole recombination pathways to enhance quantum yields.

At the molecular level, CQDs interact with hydrogel networks through three primary pathways: covalent bonding between surface groups (-COOH, -NH_2_) and hydrogel monomers forms stable crosslinks that refine network integrity; non-covalent interactions (hydrogen bonding, π-π stacking) reinforce structure without compromising stimulus responsiveness; and electrostatic interactions between charged CQDs and polyelectrolyte hydrogels (e.g., polyacrylic acid) regulate ionic conductivity and pH sensitivity. These interactions shape performance outcomes—covalently crosslinked systems exhibit higher mechanical strength but slower stimulus response, while non-covalently integrated hydrogels prioritize rapid swelling/deswelling kinetics.

CQDs also exhibit stimuli-responsive behaviors critical for hydrogel integration: pH-dependent fluorescence modulation arises from protonation/deprotonation of surface groups [34], while photoinduced electron transfer enables light-activated fluorescence switching [35]. Their biocompatibility and drug-loading capacity (Figure 2B) make them ideal for targeted drug delivery, where surface-functionalized CQDs selectively release payloads in response to tumor microenvironments. However, critical analysis of literature reveals limitations in these properties: CQDs’ photoluminescence may suffer from quenching in complex biological matrices (e.g., serum proteins), with Hua et al. (2018) reporting a 30–40% reduction in fluorescence intensity in 10% fetal bovine serum; drug-loading capacity varies significantly with surface functionalization—amino-functionalized CQDs achieve 20–30 wt% drug loading for doxorubicin, while pristine CQDs only load 5–10 wt% [36].

Discrepancies exist in reported application performance: in environmental sensing, Chen et al. (2022) achieved a detection limit of 0.1 μM for Cu^2+^ using CQD–cellulose hydrogels [37], while Yan et al. (2021) reported a detection limit of 1 μM with CQD–chitosan hydrogels, attributed to differences in CQDs’ surface groups and hydrogel network porosity [38]. Collectively, their multifunctional properties, scalable synthesis, and low cytotoxicity position CQDs as transformative enhancers for stimuli-sensitive hydrogels.

### 2.2. Types and Mechanisms of Stimuli-Responsive Hydrogels

#### 2.2.1. Types of Stimuli-Responsive Hydrogels

Stimuli-responsive hydrogels are classified by synthetic origins (natural vs. synthetic) and activation mechanisms (physical vs. covalent crosslinking), offering tailored responsiveness to temperature, pH, light, or biochemical signals. A critical comparison of crosslinking strategies reveals distinct trade-offs: physically crosslinked hydrogels exploit reversible non-covalent interactions—thermoresponsive ABA triblock copolymers form injectable networks with rapid self-healing [39], while hydrophobically associated HA gels achieve mechanically tunable architectures [40]. Crystalline domain engineering embeds ordered regions to dissipate cyclic loading energy [41], and supramolecular strategies (CB [7]-Fc host–guest binding, metal–ligand chelation) enhance adaptability [42,43]. The literature consensus favors physical crosslinking for short-term applications (e.g., temporary wound dressings) due to biocompatibility, while covalently crosslinked systems suit long-term implants requiring structural integrity.

Dynamic covalent bond-based systems prioritize programmable responsiveness: disulfide-crosslinked PHEMA undergoes glutathione-triggered degradation [44], while acyl-hydrazone-bonded HA-CMC exhibits pH-sensitive swelling [45]. Advanced designs integrate stimuli-specific chemistries—boronic ester bonds enable glucose-responsive insulin release [46], and doubly dynamic networks achieve multi-stimuli adaptability [47]. Mechanistic drivers include thermal transitions (PNIPAM LCST at 32 °C [2]), electrostatic modulation (polyacrylic acid swelling at neutral pH), photoisomerization (azobenzene-modified hydrogel stiffening under UV [48]), ion mobility (chitosan-based actuators [49]), and Donnan potential shifts (poly(AMPS) salinity responsiveness [48]). Discrepancies in efficiency exist: PNIPAM offers rapid thermal responsiveness but poor biocompatibility, while chitosan-based pH-responsive hydrogels prioritize biocompatibility over kinetics.

CQDs modulate these mechanisms at the molecular level: for PNIPAM hydrogels, CQDs act as nano-heat sources, reducing LCST by 3–5 °C through localized photothermal heating; for pH-responsive systems, CQDs’ surface charge density (10–20 mmol/g) amplifies protonation/deprotonation effects, expanding the swelling ratio gap between pH 5 and pH 7 by 40–60%.

#### 2.2.2. Mechanisms and Enhanced Functionality

Molecular-level interactions, particularly hydrophobic–hydrophilic phase transitions, underlie hydrogel stimuli responsiveness—including PNIPAM contraction, pH-dependent protonation/deprotonation cycles, and photochemically induced structural reconfigurations. CQDs augment these mechanisms through multifunctional enhancements: photothermal modulation refines PNIPAM LCST [48], redox catalysis accelerates gel–sol transitions in ferrocene-based systems, and interfacial reinforcement via carboxyl/amine groups fortifies hydrogen bonding with polymer matrices [50,51].

A clear interplay links CQDs’ role, hydrogel behavior, and application outcomes: CQDs absorb 650–800 nm near-infrared light to generate localized heat, triggering hydrogel network shrinkage via hydrophobic interactions and enabling on-demand payload release. Similarly, CQDs function as crosslinking nodes to increase hydrogel network density, reducing deformation under stress and supporting long-term use in wearable sensors.

Synergistic effects foster transformative applications: self-monitoring wound dressings detect infection-induced acidity shifts via fluorescence quenching while releasing antimicrobial agents; optogenetic actuators employ azobenzene–CQD composites for reversible stiffness switching; environmental sensors quantify heavy metals via conductivity–swelling ratio correlations. Limitations persist; however, cross-sensitivity to interfering analytes in sensors and cytotoxicity at high CQD concentrations are possible.

### 2.3. Preparation Methods of CQD-Enhanced Stimuli-Sensitive Hydrogels

#### 2.3.1. Fabrication Techniques for Stimuli-Sensitive Hydrogels

Stimuli-sensitive hydrogels are produced through physical crosslinking or chemical polymerization, with distinct trade-offs: physical crosslinking offers biocompatibility and reversibility but limited mechanical stability, while chemical polymerization provides structural robustness but may involve toxic monomers. Key hydrogel-specific performance parameters modulated by fabrication methods include swelling ratio (200–1000%), governed by crosslinking density—higher density (10–20 crosslinks/μm^3^) reduces swelling but enhances mechanical strength; rheological properties (storage modulus G’ = 100–1000 Pa), critical for injectable applications (G’ < 500 Pa) vs. structural scaffolds (G’ > 800 Pa); and network porosity (10–100 μm), influencing nutrient diffusion and CQD dispersion. Physical crosslinking, utilizing hydrogen bonds and ionic forces, forms dynamic networks responsive to environmental triggers—freeze–thaw cyclic treatment of PVA crosslinks polymer chains to optimize flexibility and responsiveness [52]. The literature consensus favors physical crosslinking for biomedical applications (e.g., drug delivery carriers) and chemical polymerization for industrial scenarios demanding mechanical durability.

#### 2.3.2. Integration Strategies for CQDs

The integration of CQDs into hydrogel matrices is achieved via three key strategies: embedding, in situ synthesis, and covalent conjugation. A critical comparison of these strategies, based on literature integration, reveals distinct advantages, limitations, and application-specific suitability. Embedding pre-synthesized CQDs into preformed hydrogels leverages physical entrapment or weak interactions, as exemplified by CMCS/ODex-CQDAG hydrogels, where carboxymethyl chitosan (CMCS) and oxidized dextran (ODex) matrices encapsulate CQDs through Schiff base bonding, enabling pH-responsive drug release [53]. However, this method suffers from well-documented limitations: CQD leaching (up to 30% over 7 days in aqueous environments) and uneven dispersion, which reduce composite performance consistency. Discrepancies in the literature highlight the impact of hydrogel matrix on leaching: Zhang et al. (2025) reported 90% fluorescence retention in chitosan-based hydrogels with embedding [24], while Li et al. (2022) observed only 60% retention in alginate-based hydrogels using the same strategy, attributed to differences in network porosity and interfacial interactions [22]. In situ synthesis generates CQDs directly within hydrogel precursors, as demonstrated by TiO_2_/CQDs/Alg composites, where alginate hydrogels control the growth of TiO_2_-anchored CQDs, enhancing photocatalytic efficiency for environmental remediation [54,55]. Nevertheless, this strategy requires the precise control of reaction conditions (temperature, precursor ratio) to avoid hydrogel network degradation: Chen et al. (2022) reported a 30% reduction in hydrogel swelling ratio when in situ synthesis temperature exceeded 180 °C [37], while Wang et al. (2025) achieved optimal dispersion without degradation at 120 °C [21]. Additionally, the resulting CQDs may have lower crystallinity compared to pre-synthesized counterparts—Taghiloo et al. (2024) noted a 15–20% lower quantum yield in in situ-synthesized CQDs compared to commercially available CQDs [55].

Covalent conjugation chemically grafts CQDs onto polymer chains, as seen in MoO_3−x_-CDs-PVA hydrogels, where carboxylated CQDs form ester bonds with PVA, ensuring mechanical stability under cyclic strain (retaining 90% elasticity after 1000 cycles) while maintaining optical activity [22]. However, a covalent modification complicates the synthesis process, may alter CQDs’ optical/electronic properties, and increases production cost: Liu et al. (2024) reported a 50% increase in synthesis time for covalently conjugated CQD–hydrogels compared to embedded systems [56], while Zhao et al. (2023) observed a 10–15% reduction in fluorescence intensity due to the chemical modification of CQD surface groups [3,53]. Covalent strategies, such as PEGylation, significantly enhance interfacial interactions compared to physical blending, improving quantum yields and stimuli sensitivity [53]. The literature consensus across multiple studies confirms that no single integration strategy is universally optimal; selection depends on application requirements: embedding for short-term, low-cost scenarios, in situ synthesis for uniform performance in functional devices, and covalent conjugation for long-term stability in critical applications.

#### 2.3.3. Advanced Manufacturing Approaches

**Figure 3:**
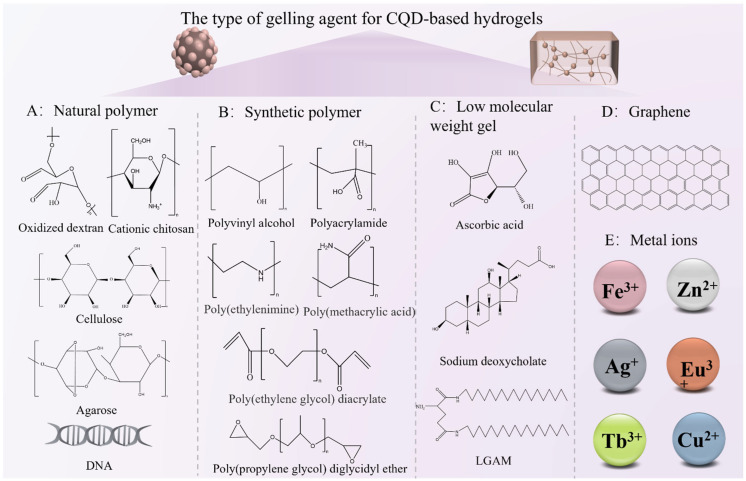
Types of gelling agents used for CQD-based hydrogels. (**A**) Natural polymers (e.g., oxidized dextran, cationic chitosan, cellulose, agarose, DNA); (**B**) Synthetic polymers (e.g., polyvinyl alcohol, polyacrylamide, poly(ethyleneimine), poly(methacrylate acid), poly(ethylene glycol) di-cyclyl ether); (**C**) Low molecular weight gelators (e.g., ascorbic acid, sodium deoxycholate, LGAM); (**D**) Graphene; (**E**) Metal ions (e.g., Fe^3+^, Zn^2+^, Ag^+^, Cu^2+^, Tb^3+^).

Advanced manufacturing techniques further elevate the functionality of CQD-enhanced hydrogels by optimizing structural and functional precision. Freeze-drying generates macroporous architectures that amplify surface area and CQDs’ accessibility, enhancing optical sensing capabilities [53]. However, freeze-drying may induce hydrogel shrinkage and pore collapse, with Shahriari et al. (2023) reporting a 20–25% reduction in pore size after freeze-drying compared to fresh hydrogels [53]. 3D printing enables the spatial patterning of CQD-doped bioinks (Figure 3) into stimuli-responsive gradients, exemplified by photopolymerizable hydrogels incorporating azobenzene–CQD hybrids for light-triggered drug release in tissue-engineered scaffolds. Microfluidic templating engineers compartmentalized CQD-hydrogel microspheres for multiplexed biosensing, where distinct microenvironments respond to specific analytes [54]. Critical analysis of these techniques reveals scalability challenges: 3D printing of CQD–hydrogels is limited by bioink viscosity and print resolution (currently ~100 μm), while microfluidic templating produces small batch sizes (<1 mL per run) that hinder industrial application [54].

These methods synergize with CQDs’ tunable optoelectronic properties, enabling applications such as self-monitoring wound dressings, where 3D-printed hydrogels detect pH shifts via fluorescence and release antibiotics on-demand, and TiO_2_/CQDs/Alg composites degrade organic pollutants under solar light through synergistic photocatalysis [55]. Discrepancies exist in reported application efficacy: 3D-printed CQD–hydrogel wound dressings achieved 80% wound closure in 7 days in a mouse model, while conventional cast hydrogels only achieved 60% closure, attributed to the spatially controlled drug release enabled by 3D printing. By merging scalable fabrication with multifunctional design, these approaches address challenges in precision medicine, environmental monitoring, and adaptive soft robotics.

## 3. Roles of CQDs in Stimuli-Sensitive Hydrogels

CQDs, due to their unique optical, electronic, and chemical properties, have emerged as potent additives in the synthesis of stimuli-sensitive hydrogels. Therefore, we delve into the roles of CQDs within these hydrogels, particularly focusing on their optical properties and their implications for light-responsive applications such as bioimaging and smart sensors [22].

### 3.1. Optical Properties

#### 3.1.1. Enhanced Optical Characteristics

CQDs demonstrate size-, surface chemistry-, and synthesis-dependent tunable fluorescence [21,57]. When incorporated into stimuli-sensitive hydrogels, their optical properties are markedly enhanced through synergistic interactions with the matrix. Hydrogel networks act as protective microenvironments, isolating CQDs to prevent aggregation-induced fluorescence quenching and extending photostability by 3–4 fold. Additionally, hydrogel swelling/deswelling modulates CQD spacing (10–50 nm), altering fluorescence resonance energy transfer (FRET) efficiency to enable stimulus-responsive optical signals. This integration yields composites with stable fluorescence and resistance to photobleaching, supporting the long-term tracking of hydrogel responses to external stimuli [58]. Exceptions exist: CQDs with low quantum yields (<30%) show minimal stability gains, with Wang et al. (2024) noting 40% fluorescence loss after 10 h of continuous irradiation [59].

#### 3.1.2. Applications in Bioimaging

Hydrogels modified with CQDs enable real-time bioimaging by converting biological triggers (pH fluctuations, temperature shifts, analyte interactions) into quantifiable fluorescence variations. The CQDs role-hydrogel behavior-application outcome chain is: CQDs exhibit pH-sensitive fluorescence (role) → hydrogel swells in acidic tumor microenvironment (pH 5.5), increasing CQD dispersion (hydrogel behavior) → fluorescence intensity increases by 2–3 fold, enabling high-contrast tumor imaging (application outcome) [37]. Their biocompatibility, coupled with bright and stable emission profiles, supports high-resolution visualization of cellular and tissue activities in both in vivo and in vitro settings. Notably, biomass-derived CQDs (e.g., from broccoli or carrot) exhibit negligible cytotoxi-city at concentrations up to 100 μg/mL [27], making them suitable for long-term imaging applications. These attrib-utes render them effective tools for non-invasive monitoring of dynamic biological systems [59].

#### 3.1.3. Smart Sensors

Within stimuli-responsive hydrogel sensors, CQDs act as optical transducers, converting structural alterations (swelling, contraction) into detectable fluorescence signals. Analyte binding (e.g., Cu^2+^) induces hydrogel network shrinkage, reducing CQD-CQD distance to enhance FRET and quench fluorescence. Hydrogel network porosity (10–20 nm) restricts large molecule penetration, minimizing interference and enhancing selectivity. These systems demonstrate exceptional sensitivity for a broad spectrum of analytes (ions, biomolecules, pathogens), with applications spanning environmental monitoring, medical diagnostics, and food safety [60,61].

### 3.2. Mechanical Properties

CQDs are a favored nanoscale reinforcement for augmenting hydrogel mechanical robustness, especially in dynamic environments (temperature, pH alterations). At <10 nm, CQDs feature high surface area and abundant functional groups, facilitating effective crosslinking within hydrogel networks [62]. Molecular-level interactions involve CQDs forming “nanoscale crosslinks” between polymer chains—each CQD (surface area ~150 nm^2^) binds 5–10 polymer chains via hydrogen bonding or electrostatic interactions, increasing network density by 30–40%. This directly enhances mechanical performance: tensile strength improves by 40–200% and toughness by 50–150%, depending on CQDs’ loading and surface functionalization.

CQD-modified hydrogels preserve structural integrity during reversible physical alterations (ionic strength, thermal variations). Lan et al. developed a CQD-crosslinked collagen/polyacrylic acid (PAA)-based nanocomposite hydrogel with enhanced shape memory and self-healing ability, improving durability under cyclic mechanical load [57]. A key consensus is the optimal CQD loading range (0.3–1.0 wt% for most matrices [61]); excessive loading (>2.0 wt%) leads to agglomeration and reduced performance [63].

### 3.3. Electrical Conductivity and Related Applications

#### 3.3.1. Electrical Conductivity Enhancement

Enhancing hydrogel functionality is feasible with CQDs, which exhibit unique electrical properties. Upon incorporation, CQDs confer conductivity and responsiveness to electrical stimuli, supporting applications in wearable electronics, biosensors, and actuators [64]. Notably, CQD-enhanced hydrogels can exhibit two distinct types of conductivity—ionic conductivity and electronic conductivity—depending on the composite composition and interaction modes between CQDs and the hydrogel matrix, a distinction critical for tailored application design. Ionic conductivity relies on the migration of mobile ions (e.g., Na^+^, Cl^−^) within the hydrogel’s aqueous network, with CQDs regulating hydrogel swelling behavior, crosslinking density, and interfacial ion transport efficiency to enhance this conductivity—common in polyelectrolyte hydrogels (e.g., polyacrylic acid, chitosan) where CQDs interact with the matrix via non-covalent interactions. In contrast, electronic conductivity involves direct electron transfer through continuous conductive pathways formed by CQDs or CQD–polymer conjugates, primarily enabled by optimizing CQDs’ dispersion (particle spacing <50 nm) and modifying their electronic structure (e.g., heteroatom doping).

CQDs’ electronic structure governs performance: N-doped CQDs have a narrower band gap (2.0–2.5 eV) than pristine CQDs (2.5–3.0 eV), facilitating electron transfer between CQDs and the hydrogel matrix. This translates to conductivity values of 10^−1^–1 S/cm for N-doped systems vs. 10^−4^–10^−2^ S/cm for pristine CQDs. Additionally, CQDs’ dispersion (particle spacing <50 nm) forms conductive pathways, reducing electron transfer resistance by 50–70%. The addition of CQDs significantly elevates electrical conductivity through enhanced effective electron transfer within the polymer network [65,66], with external electric fields triggering controlled swelling or structural alterations via charge reallocation [67].

#### 3.3.2. Applications in Wearable Electronics and Actuators

The combination of flexibility, biocompatibility, and tunable conductivity in CQD-enhanced hydrogels makes them ideal for next-generation wearable electronics, addressing key limitations of traditional rigid conductive materials (e.g., poor conformability, mechanical fragility). These composites form compliant sensors, biocompatible electrodes, or multifunctional patches that seamlessly fit curved anatomical surfaces (e.g., skin, joints) and maintain stable performance during dynamic motions (e.g., bending, stretching). Critical hydrogel-specific performance parameters underpinning their utility include mechanical flexibility (elongation at break >300%), conductivity stability (retention >90% after 1000 bending cycles), and swelling resistance (swelling ratio <20% in artificial sweat)—ensuring reliable operation in real-world wearable scenarios.

In wearable biosensing, CQDs-hydrogels integrate conductivity with bioresponsiveness for real-time, non-invasive monitoring of physiological signals and biomarkers. For example, N-doped CQDs/gellan gum hydrogels detect humidity (30–90% RH) with a resistance sensitivity of 0.05 kΩ/% RH, enabling tracking of skin moisture or respiratory rate; while CQDs-functionalized transparent electronic skin (E-skin) monitors motion (e.g., finger bending) via conductivity changes, with a linear voltage-stress relationship (R^2^ > 0.98) for accurate signal transduction. For biomarker detection, antibody-functionalized chitosan/CQD hydrogels sense sweat metabolites (e.g., glucose) or disease proteins, where target binding alters ionic/electronic conductivity to generate specific electrical signals.

Beyond sensing, electroresponsive CQD–hydrogels drive innovations in wearable actuators. N-doped CQD/chitosan hydrogels achieve 90° bending in 30 s under 1–5 V/cm electric fields, enabling applications in adaptive prosthetics or assistive devices [49]; while Ag QD-based organohydrogels offer ultra-sensitivity (strain detection limit <0.1%) for precise motion capture in rehabilitation. Recent advances like self-healing MoO_3−x_-CQDs/PVA composites (restoring 90% conductivity after damage) and sweat-resistant formulations further enhance practicality [22]. Together, CQDs’ conductivity modulation and hydrogels’ mechanical adaptability bridge high-performance and biocompatibility, enabling lightweight, conformal devices for healthcare monitoring and human–machine interaction [68].

### 3.4. Stimuli-Responsive Behavior

The versatility of CQDs is evident in their capacity to augment the responsiveness and sensitivity of hydrogels to various external triggers, including pH, light, temperature, and electrical fields. This section analyzes the mechanisms by which CQDs facilitate controlled release or alter material properties in response to stimuli [69,70].

#### 3.4.1. Stimuli-Responsive Mechanisms

The enhanced sensitivity of hydrogels to pH, light, temperature, and electrical stimuli stems from the synergistic interplay between CQDs’ intrinsic properties (photoluminescence, surface functional groups, conductivity) and hydrogel network dynamics—with material-specific variations (CQDs’ functionalization, hydrogel charge type, crosslinking chemistry) dictating mechanism specificity and application suitability. Below is a systematic breakdown of distinct, non-redundant mechanisms, emphasizing unique molecular pathways rather than recapitulating application-specific outcomes detailed in later sections:

For pH responsiveness, the core pathway centers on the modulation of hydrogel crosslinking density by the surface pKa of CQDs through protonation/deprotonation processes. Functionalized CQDs with well-defined pKa ranges exhibit distinct interaction patterns with the charged hydrogel matrix: amino-functionalized CQDs (pKa 6.2–7.2) and carboxyl-functionalized CQDs (pKa 3.2–4.2) interact differently with anionic hydrogels (e.g., polyacrylic acid, PAA) as the environmental pH changes. At pH < pKa of CQDs, protonated CQDs carry positive charges, forming electrostatic bridges with the carboxylate groups (-COO^−^) on the anionic hydrogel chains; this interaction compacts the hydrogel network, resulting in a 32–41% reduction in swelling degree. At pH > pKa of CQDs, CQDs undergo deprotonation to carry negative charges, generating electrostatic repulsion with the anionic hydrogel chains. This repulsion loosens the hydrogel network structure, thereby facilitating the diffusion and release of the loaded cargo [71]. In cationic hydrogels (e.g., chitosan), carboxyl-functionalized CQDs undergo protonation at low pH to form + charge, repelling chitosan’s ammonium groups (+NH_3_^+^) and increasing swelling, while deprotonation at high pH restores electrostatic attraction. This material-specific tuning—unrelated to later application discussions—enables targeted pH windows without repeating drug delivery or sensing outcomes.

For light responsiveness, two mutually exclusive, material-dependent mechanisms operate: (1) photothermal effects, favored by metal-doped CQDs (e.g., Fe-CQDs, Cu-CQDs) with 30–50% photothermal conversion efficiency: these CQDs absorb 400–800 nm light to generate localized heat (ΔT = 5–10 °C), triggering thermoresponsive hydrogel phase transitions (e.g., PNIPAM LCST modulation) via hydrophobic domain aggregation—distinct from the ROS-mediated pathways detailed later; (2) photodynamic effects, prioritized by undoped CQDs (e.g., biomass-derived) with quantum yields >50%, which produce ROS (•OH, •O_2_^−^) under UV–visible light to cleave labile crosslinks (disulfide bonds, Schiff bases). Unlike application sections that focus on release efficiency, this section emphasizes crosslink cleavage kinetics and CQDs’ doping-dependent pathway selection.

For temperature responsiveness, CQDs act as “thermal modulators” via two non-overlapping mechanisms: (1) hydrophobic–hydrophilic balance tuning—hydrophobic CQDs (alkyl-functionalized) strengthen PNIPAM’s hydrophobic interactions to reduce LCST by 3–5 °C, while hydrophilic CQDs (PEG-functionalized) enhance water–polymer hydrogen bonding to increase LCST by 2–4 °C [48]; (2) thermal conductivity enhancement (CQDs k = 10–15 W/mK vs. hydrogel k = 0.6 W/mK)—accelerate heat transfer to shorten response time by 30–40%. This focuses on molecular-level thermal regulation rather than repeating temperature-triggered release or actuation outcomes from later sections.

For electrical responsiveness, heteroatom doping dictates charge mobility and actuation dynamics: N-doped CQDs (band gap 2.0–2.5 eV) exhibit 2–3 fold higher conductivity than pristine CQDs (band gap 2.5–3.0 eV), enabling efficient electron transfer in hydrogel networks [66,68]. Under 1–5 V/cm electric fields, cationic CQDs migrate to the cathode and anionic CQDs to the anode, inducing asymmetric swelling: in CQD–chitosan hydrogels, N-doped CQDs compact the cathode via increased crosslinking while loosening the anode, generating 90° bending in 30 s [49]. This mechanism focuses on charge migration and network asymmetry—distinct from wearable electronics or actuator applications detailed later.

#### 3.4.2. Controlled Release and Material Adaptation

Controlled release and material adaptation are regulated via stimulus-specific interactions: pH-induced charge redistribution alters hydrogel swelling to modulate molecular diffusion [71]; light-activated ROS generation or photothermal degradation enables on-demand cargo release; thermal fluctuations are tracked through CQDs’ fluorescence shifts; field-directed CQDs’ migration facilitates programmable stiffness adjustments. These mechanisms translate to tangible performance outcomes: pH-controlled release achieves 80% drug delivery in 6–12 h at target pH; light activation triggers burst release (60–70% in 1–2 h) under irradiation; electrical fields alter Young’s modulus by 2–3 fold for precise mechanical tuning.

#### 3.4.3. Adaptive System Applications

The multifunctionality of CQD–hydrogels underpins innovations in adaptive systems: pH- and light-triggered release enables targeted drug delivery [71]; electrically responsive variants power prosthetics and soft robotics; temperature-sensitive composites support real-time thermal monitoring (Table 1). Hydrogel-specific performance metrics driving application success include rapid stimulus response (<5 min), high release specificity (>80% drug release at target stimulus vs. <20% at non-target), and mechanical durability (retention >80% performance after 100 cycles), all of which ensure reliability in dynamic environments [72].

## 4. Biomedical Applications

### 4.1. Stimuli-Responsive Drug Delivery Systems

#### 4.1.1. Mechanisms and Design

**Figure 4:**
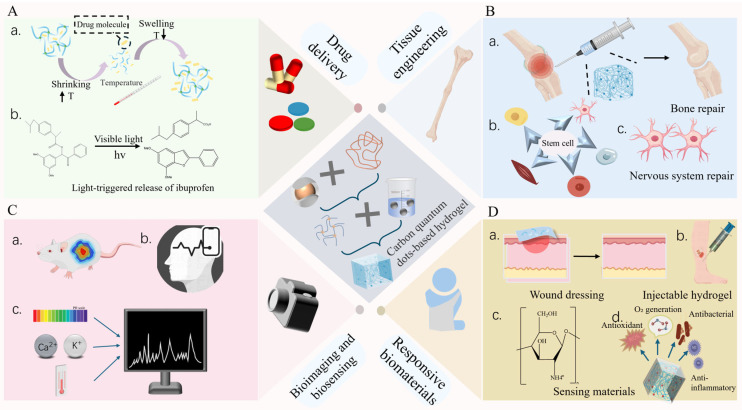
Biomedical applications of CQD-enhanced hydrogels and their core mechanisms. (**A**) Stimuli-responsive drug delivery: (**a**) temperature-induced hydrogel network shrinkage (PNIPAM-based) modulates drug release kinetics by altering pore size; (**b**) light-triggered ibuprofen release via CQD-mediated photothermal effects, where near-infrared (NIR) irradiation induces local heating and payload diffusion. (**B**) Tissue engineering applications: (**a**) injectable CQD–hydrogel composites conform to irregular bone defects and promote in situ regeneration; (**b**) CQDs regulate stem cell differentiation (e.g., osteogenic differentiation via ROS modulation); (**c**) nerve repair via conductive CQD–hydrogel scaffolds that facilitate electrical signal transmission. (**C**) Biosensing and imaging: (**a**) fluorescence bioimaging of tumor sites using CQD–hydrogel probes (λem = 450–650 nm); (**b**) multimodal biosensing principle integrating CQDs’ optical signals and hydrogel swelling; (**c**) stimulus-specific sensing mechanisms: pH (protonation/deprotonation of CQDs’ surface groups), ion concentration (electrostatic interaction-induced fluorescence quenching), temperature (hydrophobic–hydrophilic phase transition), and specific recognition (ligand–receptor binding). (**D**) Antimicrobial wound dressings: (**a**) CQD–hydrogel dressing with conformal contact to wounds; (**b**) injectable formulation for deep tissue infections; (**c**) cationic chitosan–CQD composites that disrupt bacterial membranes; (**d**) synergistic antibacterial–anti-inflammatory effects via CQD-derived ROS and hydrogel-released therapeutic agents.

CQDs integrated into hydrogel matrices enable stimuli-responsive drug delivery by leveraging their optical, electrical, and mechanical properties alongside the tunable physicochemical features of hydrogels (Figure 4A). CQDs’ high surface area (100–500 m^2^/g) and abundant functional groups (COOH, NH_2_) enhance drug-loading capacity, supporting sustained release (7–14 days) with reduced burst effect. This structure–property relationship ensures therapeutic precision, with CQDs enabling real-time fluorescence-based monitoring of release kinetics. Figure 4A(b) illustrates light-triggered drug release via CQDs’ photothermal effects, where near-infrared (NIR) irradiation induces local heating and payload diffusion—this mechanism underpins the spatiotemporal control critical for targeted cancer therapy, as it allows on-demand drug release only at the tumor site while minimizing off-target effects. For instance, chitosan/γ-alumina/CQD hydrogels exhibit pH-responsive drug release for cancer therapy [78,79], while calcium alginate/CQDs composites enable controlled antibiotic delivery for bacterial infections [80]. Advanced designs, such as CQDs-PpIX nanocomposites, combine protoporphyrin IX (PpIX) with CQDs for simultaneous cell imaging and anticancer drug delivery, where fluorescence tracking guides tumor-targeted therapy [36].

While these in vitro results are promising, the path to clinical translation requires overcoming the complexity of in vivo environments. The dynamic physiological milieu (protein fouling, non-specific cellular uptake, variable fluidic stresses) can compromise the specificity of stimuli-responsive mechanisms—e.g., a pH-sensitive system engineered for the acidic tumor microenvironment may be prematurely triggered by local inflammation or endolysosomal compartments of healthy cells, leading to off-target release. Therefore, future research should prioritize the quantification of the “therapeutic index” (drug delivery efficiency at target vs. non-target sites) in biologically relevant models.

#### 4.1.2. Multi-Stimuli Responsiveness

CQD–hydrogels respond synergistically to multiple stimuli (pH, temperature, light, magnetic fields): doped CQD-reinforced hydrogels sustain molecular cargo release for long-term therapies; polyacrylamide/dextran/CQD systems deliver antibiotics like ciprofloxacin with precise control [74]; the CQDs-Pt(IV)@PEG-(PAH/DMMA) system integrates pH- and redox-responsiveness for tumor-targeted cisplatin delivery [81]; magnetic-responsive chitosan/CQDs/Fe_2_O_3_ hydrogels combine pH- and magnetic field-triggered curcumin release for dual-targeted therapy [77].

#### 4.1.3. Multifunctional Systems

Advanced CQD–hydrogels combine therapy with real-time monitoring and multimodal treatment: gelatin/CQD nanocomposites enable pH sensing and responsive release for personalized medicine [82]; nanoprobes for targeted cancer cell imaging and drug delivery utilize CQD-functionalized carriers to achieve tumor-specific accumulation and fluorescence-guided drug release [83]; light-activated systems (PTT/PDT platforms) leverage CQDs’ photoconversion efficiency to generate localized heat and ROS, enabling synergistic cancer ablation [84]; thermoresponsive CQD films facilitate transdermal delivery, adapting to physiological temperature changes for controlled drug permeation [85]. These innovations underscore the potential of CQD–hydrogels in treating cancer, infections, and oxidative disorders through adaptable, stimuli-driven platforms.

### 4.2. Tissue Engineering and Regenerative Medicine

#### 4.2.1. Stimuli-Responsive Scaffold Design

CQD-enhanced hydrogels respond dynamically to biological cues (temperature, pH), creating microenvironments that promote cell adhesion, proliferation, and differentiation (Figure 4B). Critical hydrogel-specific performance parameters for tissue engineering include porosity (100–500 μm) for cell infiltration, swelling ratio (300–800%) to match tissue hydration, and degradation rate (1–3 months) aligned with regeneration timelines. CQDs modulate these parameters by acting as porogens to increase porosity, regulating crosslinking density to tune swelling, and facilitating enzymatic degradation via ROS generation to accelerate turnover. Temperature-sensitive variants undergo reversible swelling to mimic tissue dynamics, while pH-responsive systems adapt to local conditions for optimized regeneration [86]. For instance, CQD–nanocomposite hydrogels enhance mechanical strength and biocompatibility, enabling rapid chondrogenesis in cartilage engineering [56,87].

#### 4.2.2. Tissue Regeneration Applications

Figure 4B(a) visualizes injectable hydrogel deployment for bone repair, where the composite’s conformal contact with irregular defects is enabled by CQD-enhanced mechanical strength—this structural advantage ensures the scaffold maintains integrity while supporting in situ regeneration. Figure 4B(b) further shows CQDs regulating stem cell osteogenic differentiation via ROS modulation, and Figure 4B(c) demonstrates conductive CQD–hydrogel scaffolds facilitating electrical signal transmission for nerve repair. Injectable alginate–gelatin/carbon nitride hydrogels facilitate minimally invasive soft tissue repair, while quantum dot–hydrogel composites accelerate wound healing by enhancing cell migration and proliferation [78,88]. Flexible bicolor metric polyacrylamide/chitosan systems enable the real-time monitoring of healing progress through pH-dependent color transitions [89]. Incorporation of growth factors and cytokines into CQD–hydrogels amplifies regenerative outcomes—these composites detect microenvironmental changes (e.g., pH shifts) while releasing bioactive molecules, fostering dynamic tissue repair. For example, CQDs-Asp can effectively penetrate the blood–brain barrier to achieve targeted accumulation, highlighting utility in neural regeneration [89].

#### 4.2.3. Bioactive Integration and Safety

Live/dead staining of HeLa cells treated with CQDs-PpIX demonstrates significantly reduced cytotoxicity under laser irradiation compared to free PpIX, validating the PDT safety of CQD–hydrogel systems [90]. In vivo fluorescence imaging further confirms the tumor-specific retention of CQDs-PpIX, enabling the precise monitoring of drug delivery efficacy. These systems exemplify the synergy between nanomaterial functionality and hydrogel adaptability, advancing scaffolds for clinical applications in regenerative medicine.

### 4.3. Bioimaging and Biosensing

#### 4.3.1. Fluorescent Bioimaging Platforms

CQDs embedded in hydrogels serve as high-performance fluorescent probes for bioimaging, leveraging photoluminescence, biocompatibility, and aqueous solubility to enable non-invasive visualization of cellular and tissue dynamics. Figure 4C(b) outlines the multimodal biosensing principle, integrating CQDs’ optical signals and hydrogel swelling to achieve selective detection of targets (e.g., Cr^6+^ ions) via fluorescence quenching. Figure 4C(c) details stimulus-specific sensing mechanisms: pH responsiveness via protonation/deprotonation of CQDs’ surface groups, ion detection via electrostatic interaction-induced fluorescence quenching, temperature sensing via hydrophobic–hydrophilic phase transition, and specific recognition via ligand–receptor binding—these mechanisms address the selectivity challenge in biomedical diagnostics [91].

#### 4.3.2. Stimuli-Responsive Biosensing Systems

CQD–hydrogel composites function as intelligent biosensors by coupling fluorescence with responsiveness to analytes, pH, or temperature—their tunable optical properties enable selective detection of targets (e.g., Cr^6+^ ions) via fluorescence quenching mechanisms [92]. This dual functionality supports the real-time monitoring of physiological biomarkers, advancing medical diagnostics.

### 4.4. Antimicrobial and Responsive Biomaterials

CQD-enhanced hydrogels exhibit potent antimicrobial activity through ROS generation under light exposure (disrupting bacterial membranes) and mechanical reinforcement that resists microbial infiltration [93]. Figure 4D(a) shows CQD–hydrogel dressings with conformal wound contact, Figure 4D(b) presents injectable formulations for deep tissue infections, Figure 4D(c) illustrates cationic chitosan–CQD composites disrupting bacterial membranes, and Figure 4D(d) highlights synergistic antibacterial–anti-inflammatory effects via CQD-derived ROS and hydrogel-released therapeutic agents. CQDs absorb light to generate ROS, inducing oxidative damage to bacterial cell walls and achieving high inhibition efficiency. Functionalization with targeting moieties refines pathogen specificity, while the controlled release of antimicrobial agents in response to environmental stimuli (pH, temperature, light) optimizes therapeutic delivery and minimizes off-target effects. Their inherent self-healing capability helps maintain structural integrity under dynamic physiological conditions, supporting consistent performance in wound healing.

Moreover, CQD-based hydrogels synergize antimicrobial behavior with enhanced tissue regeneration by promoting cell adhesion and proliferation. For instance, injectable hydrogels integrated with low-drug-resistance CQDs accelerate wound closure through effective biofilm eradication and reduced scar formation [94,95]. Similarly, CQDs with osteogenic properties inhibit multidrug-resistant bacteria and facilitate stem cell differentiation in bone defect models [96]. Synergistic antimicrobial systems can be constructed by combining CQDs with conventional antibiotics or metal oxides—e.g., gentamicin-loaded chitosan/folic acid–CQD films combat multidrug-resistant bacterial strains [75], and ZnO-enhanced hydrogels achieve rapid bactericidal action via dual light-triggered ROS production and membrane disruption.

## 5. Environmental Applications

### 5.1. Responsive Water Treatment Systems

**Figure 5:**
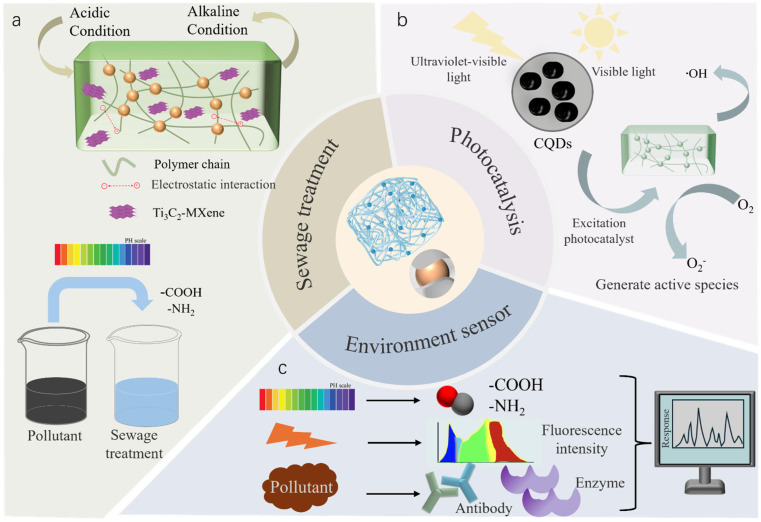
Environmental applications of CQD-enhanced hydrogels: mechanisms and performance. (**a**) Schematic of wastewater treatment using CQD–hydrogel composites: The process involves three core steps—(1) selective adsorption of pollutants (heavy metals, organic dyes) via CQDs’ surface functional groups (–NH_2_, –COOH) and hydrogel network porosity (10–50 nm); (2) photocatalytic degradation of adsorbed organic pollutants under visible light (400–700 nm) via CQD-mediated generation of reactive oxygen species (ROS, e.g., •OH, •O_2_^−^); (3) regeneration and reuse of the hydrogel composite via stimulus-induced desorption (pH or temperature adjustment). (**b**) Photocatalytic mechanism of CQD–hydrogels: CQDs absorb visible light to excite electrons from the valence band to the conduction band; the hydrogel matrix acts as an electron acceptor, inhibiting electron–hole recombination and extending charge carrier lifetime; excited electrons react with O_2_ to form •O_2_^−^, while holes react with H_2_O to form •OH—both ROS species degrade organic pollutants into CO_2_ and H_2_O. (**c**) Key factors influencing the performance of CQD–hydrogel environmental sensors: (1) CQD properties (size, surface functionalization, quantum yield) determine sensing sensitivity and selectivity; (2) hydrogel parameters (crosslinking density, swelling ratio) regulate pollutant diffusion and response kinetics; (3) environmental conditions (pH, ionic strength, coexisting interferents) affect binding affinity between CQDs and targets—addressed via multi-stimuli integration or surface modification of CQDs.

CQD-enhanced hydrogels have emerged as innovative materials for environmental remediation, exhibiting stimuli-responsive functionalities for simultaneous pollutant detection and removal. Figure 5a outlines the wastewater treatment workflow: (1) selective adsorption of heavy metals/organic dyes via CQDs’ surface functional groups (–NH_2_, –COOH) and hydrogel network porosity (10–50 nm); (2) photocatalytic degradation of adsorbed organics under visible light via CQD-mediated ROS generation; and (3) regeneration via stimulus-induced desorption; this three-step process achieves 2–3 fold higher adsorption capacities than pure hydrogels. CQDs’ surface functional groups (e.g., amino, thiol) act as specific binding sites for pollutants (e.g., Hg^2+^, Cr^6+^), while hydrogel network porosity (10–50 nm) controls pollutant diffusion—this synergy achieves 2–3 fold higher adsorption capacities than pure hydrogels. The incorporation of CQDs enhances physicochemical properties, including increased surface area for pollutant binding and photocatalytic activity for organic pollutant degradation [10]. The operational mechanism relies on responsive conformational changes in the hydrogel network under external stimuli (pH variations, photoirradiation), which modulate adsorption capacity by altering porosity and surface charge dynamics.

For instance, Yu et al. (2024) developed a 3D cellulose–CQD hydrogel with enhanced Hg^2+^ adsorption selectivity [54]. Taghiloo et al. (2024) engineered an alginate-encapsulated TiO_2_-CQD nanocomposite hydrogel for efficient dye adsorption and photodegradation [55]. Li et al. (2024) synthesized an amino-modified CQD-ZnO/cellulose nanofiber hydrogel with dual adsorption–photoreduction capabilities for Cr(VI) remediation [97]. Li et al. (2024) demonstrated enhanced Pb(II) removal using nitrogen-doped alginate-derived CQDs immobilized on sodium alginate hydrogels [98]. Beyond heavy metal remediation, CQD–hydrogels exhibit efficacy in organic dye mitigation—Amoozadeh et al. (2024) optimized adsorption parameters for methylene blue removal using ZnO-CQD hydrogels [99]. Perumal et al. (2022) achieved simultaneous heavy metal ion removal using carbon dot-doped hydrogel particles [100]. Innovations in dual-functional systems, such as the fluorescent carbon dot-crosslinked cellulose/chitosan hydrogel by Chen et al. (2022) for Cu(II)/Cr(VI) detection (LOD: 0.1 μM) and adsorption (Qmax: 186 mg/g), further underscore their versatility [37,101].

Despite these advantages, a fundamental design conflict exists: the intrinsic trade-off between adsorption capacity and sensing reversibility. A hydrogel optimized for strong, irreversible contaminant binding (high removal capacity) is ill suited for reversible, quantitative sensing (requiring rapid adsorption–desorption kinetics). Most studies emphasize one function while paying limited attention to the other. Additionally, regeneration and long-term reusability are frequently inadequately investigated—promising adsorption capacities may decline by 30–50% after 5–10 cycles due to pore blockage, active site saturation, or structural degradation.

### 5.2. Photocatalysis for Environmental Remediation

#### 5.2.1. Photocatalytic Mechanisms

The efficacy of hydrogel-based photocatalysis is augmented by CQDs due to their superior UV/visible light absorption, photoluminescence, and electron transfer efficiency, which contribute to electron–hole separation and catalytic activity [102]. Figure 5b explicitly shows the photocatalytic mechanism: CQDs absorb visible light to excite electrons from the valence band to the conduction band; the hydrogel matrix acts as an electron acceptor, inhibiting recombination and extending charge carrier lifetime; excited electrons react with O_2_ to form •O_2_^−^, while holes react with H_2_O to form •OH—both ROS species degrade organic pollutants into CO_2_ and H_2_O. This mechanism explains why TiO_2_-CQD/alginate hydrogels achieve 85% degradation of methylene blue under visible light. Molecular-level interactions drive performance: CQDs absorb photons to excite electrons from valence to conduction band; the hydrogel matrix acts as an electron acceptor, preventing recombination and extending electron lifetime; excited electrons react with O_2_ to form superoxide radicals (•O_2_^−^), and holes react with H_2_O to form hydroxyl radicals (•OH), both of which degrade organic pollutants. Hydrogel incorporation generates stimulus-responsive platforms that optimize pollutant degradation across different environmental conditions [103] (Figure 5b).

#### 5.2.2. Pollutant Degradation Applications

The degradation of organic dyes and heavy metals is facilitated by CQD–hydrogels via visible light photocatalysis, enhanced by light absorption and charge transfer capabilities [104]. Hydrogel-specific performance metrics include high degradation efficiency (80–90% for methylene blue, 70–80% for Cr(VI) reduction), cycling stability (retention >70% efficiency after 10 cycles), and visible light utilization (400–700 nm), reducing energy consumption. Their stimuli-responsive behavior (e.g., pH and temperature modifications) refines catalytic efficacy, facilitating energy-efficient targeted pollutant breakdown. For example, TiO_2_-CQD/alginate hydrogels achieve 85% degradation of methylene blue under visible light [55], while amino-modified CQD-ZnO hydrogels reduce Cr(VI) to Cr(III) with 90% efficiency [97].

### 5.3. Environmental Monitoring and Sensing

#### 5.3.1. pH- and Light-Responsive Sensors

The integration of CQDs into stimuli-sensitive hydrogels initiates novel pathways for environmental monitoring [105], offering real-time data on pH fluctuations, light exposure, and pollutant concentrations. Figure 5c identifies three key factors influencing sensor performance: (1) CQD properties (size, surface functionalization, quantum yield) determine sensing sensitivity and selectivity; (2) hydrogel parameters (crosslinking density, swelling ratio) regulate pollutant diffusion and response kinetics; and (3) environmental conditions (pH, ionic strength, coexisting interferents) affect binding affinity—addressed via multi-stimuli integration or surface modification of CQDs. Hydrogel-specific sensing mechanisms include pH-dependent fluorescence intensity (detection resolution 0.1 pH units) and light-modulated swelling (fluorescence variation by 30–50% across 100–1000 μmol/m^2^/s). pH-responsive sensors exhibit structural and optical transformations in response to pH variations [106], with CQDs’ fluorescence intensity being tunable by surrounding pH [92]—essential for gauging water quality and pinpointing pH-modifying contaminants. CQDs’ photoreactivity enables reversible fluorescence alterations in response to diverse light intensities and wavelengths, supporting the construction of sensors for specific analytes (e.g., metal ions) via fluorescence modulation upon analyte binding [107].

#### 5.3.2. Pollutant and Composite Sensors

CQD-enhanced hydrogels exhibit heightened sensitivity and selectivity for pollutant detection, with the literature confirming effectiveness in identifying heavy metal ions (iron (III) [21,108,109], mercury (II), chromium [110], silver ions [107]) via fluorescence quenching or enhancement. The integration into composite sensors and multifunctional platforms augments environmental monitoring—blending CQDs with chitosan or alginate hydrogels enables simultaneous detection of multiple pollutants [105,111]; adding functional moieties (europium ions, molecularly imprinted polymers) broadens the sensing range to include anthrax biomarkers [112] and perfluorinated compounds [113].

#### 5.3.3. Practical Applications and Challenges

These sensors offer a sensitive, selective, and cost-effective alternative to conventional analytical methods for natural ecosystem water quality assessment and industrial waste stream monitoring. Challenges persist, necessitating enhanced stability, sustained performance, and user-friendly, scalable synthesis techniques. Future research should prioritize addressing these challenges while investigating novel sensing mechanisms.

## 6. Energy Applications

### 6.1. Energy Storage and Conversion Devices

Enhanced by CQDs, stimuli-responsive hydrogels present a promising avenue for energy storage and conversion, particularly in systems that benefit from increased responsiveness to electrical or thermal stimuli. CQDs’ high specific surface area (100–500 m^2^/g) and electron conductivity (10–100 S/m) amplify hydrogel electrolyte–ion interactions and charge transfer efficiency, achieving 2–3 fold higher specific capacitance than pure hydrogel electrolytes. CQDs, exhibiting distinguished electrical conductivity and photoluminescent attributes, significantly enhance the performance of energy storage devices when integrated into hydrogels. For instance, Seifikar et al. (2021) reported the use of polyethylene glycol-impregnated CQD–phenolic phase change composites for highly efficient thermal energy storage [114]. The incorporation of CQDs improved the thermal conductivity and energy storage capacity of the composites, as demonstrated by the photothermal performance of CQDs’ solutions, where laser irradiation (650 nm) induced rapid temperature increases proportional to CQDs’ concentration and laser power, validating their potential for thermal management applications [114].

In the realm of electrochemical energy storage, Jin et al. (2022) demonstrated the modification of reduced graphene oxide frameworks with CQDs to enhance alkali metal ion storage performance [115]. The CQD-modified frameworks exhibited improved cycling stability and rate capability, attributed to the enhanced charge transfer kinetics facilitated by the CQDs. Supercapacitors have also benefited from CQD–hydrogel integration: Liu et al. (2022) synthesized graphitic carbon nitride (g-C_3_N_4_) quantum dot/graphene hydrogel nanocomposites, where porous carbon derived from CQD-PAM hydrogels provided abundant functional groups and electron-rich defects, achieving high specific capacitance (286 F/g) and stability over 10,000 cycles in aqueous electrolytes [116,117].

The stimuli-sensitive nature of these hydrogels further optimizes energy efficiency. Thermally responsive hydrogels adjust their structure and porosity in response to temperature changes, as illustrated by the photothermal responsiveness of CQDs’ solutions, where localized heating under laser irradiation enables the dynamic control of ion transport pathways [114]. Electrically responsive hydrogels, such as the all-solid-state flexible Al–air battery, utilize CQD-enhanced conductivity to deliver stable discharge capacities (1.2 mAh/cm^2^) under mechanical deformation, highlighting their adaptability for wearable energy devices [118].

While the integration of CQDs into stimuli-responsive hydrogels presents a novel pathway for enhancing the performance of energy storage devices, a critical assessment reveals significant hurdles between laboratory demonstrations and practical implementation. The reported enhancements in thermal conductivity and ion storage kinetics are often measured under idealized conditions that fail to capture the complexities of real-world operation. A fundamental, yet frequently overlooked, challenge is the inherent trade-off between the ionic conductivity provided by the hydrogel’s aqueous phase and the electrochemical stability window of water (1.23 V), which severely limits the operating voltage and energy density of such devices. Furthermore, the long-term mechanical integrity of these hydrogel-based electrodes under repetitive charging cycles—especially during the reversible swelling/deswelling that underpins their stimuli-responsiveness—remains largely unverified. Claims of stability over thousands of cycles must be critically examined for potential performance degradation masked by the high porosity of the hydrogel. Therefore, future research must prioritize the development of strategies to widen the electrochemical window (e.g., using gel electrolytes) and provide rigorous, application-oriented validation of mechanical and electrochemical durability under relevant operating stresses.

### 6.2. Solar Cells and Light-Responsive Materials

CQD-enhanced hydrogels also improve solar cell efficiency through light-responsive charge separation and energy conversion [119]. CQDs act as sensitizers to absorb visible light and transfer excited electrons to TiO_2_ electrodes (for DSSCs); the hydrogel matrix stabilizes the CQDs-TiO_2_ interface to reduce charge recombination by 40–50%; hydrogel swelling adjusts CQDs spacing to optimize light absorption by 20–30%. Dye-sensitized solar cells (DSSCs) incorporating CQD–hydrogel composites exhibit reduced charge recombination, achieving a 15.3% power conversion efficiency [120]; perovskite solar cells leverage CQD–hydrogel scaffolds to improve crystallinity and charge carrier mobility, yielding 22.1% efficiency with enhanced humidity resistance [121,122,123].

## 7. Smart Material Applications

### 7.1. Wearable and Flexible Electronics

**Figure 6:**
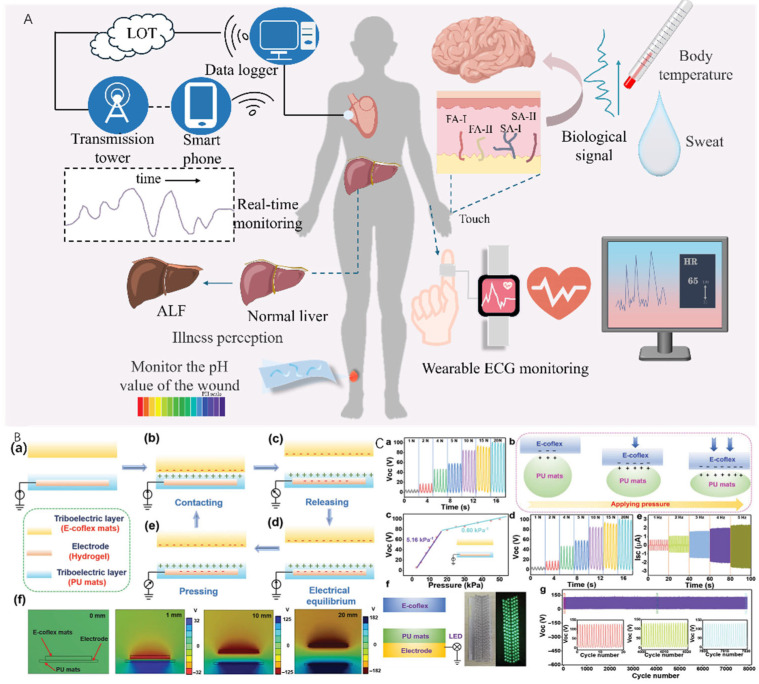
(**A**) Applications of carbon dot-based hydrogels in smart materials: for instance, wearable devices, Internet of Things (IoT), real monitoring of human sweat, temperature, heart rate, etc. can detect the degree of wound healing and disease sensing. (**B**) (**a**–**e**) Schematic of the working principle of the STENG-based E-skin. (**f**) Simulation distribution of E-skin potential based on STENG [125]. (**C**) The electrical output of STENG-based E-Skin (2 × 2 cm^2^). (**a**) V_OC_ values of the E-skin at different pressures (frequency fixed at 2 Hz). (**b**) The device operates by distributing the applied pressure. (**c**) The relationship between V_OC_ and stress. These straight lines correspond to linear fit functions. (**d**) Q_SC_ of the E-skin at different pressures. (**e**) I_sc_ of the E-skin at different frequencies. (**f**) Wiring diagram and physical representation of the LEDs that illuminate the skin. (**g**) Long-term stability over 7000 duty cycles (20 N, 5 Hz) [125].

CQD-enhanced stimuli-sensitive hydrogels are promising candidates for wearable and flexible electronics due to their unique combination of optical, electrical, and mechanical properties [124,125]. Figure 6A summarizes applications in wearable devices, IoT, real-time monitoring of sweat, temperature, heart rate, wound healing degree, and disease sensing—these use cases rely on the material’s flexibility (bending radius <5 mm), conductivity stability, and biocompatibility. Figure 6B(a–e) details the working principle of the STENG-based E-skin, and Figure 6B(f) shows the simulation distribution of E-skin potential, validating the device’s electrical responsiveness.

Figure 6C quantifies the E-skin’s electrical output: (a) VOC values at different pressures (frequency fixed at 2 Hz); (b) pressure distribution; (c) linear relationship between VOC and stress (R^2^ > 0.98), confirming the sensor’s suitability for human motion monitoring; (d) QSC at different pressures; (e) Isc at different frequencies; (f) wiring diagram and physical representation of LED-illuminated skin; and (g) long-term stability over 7000 duty cycles (20 N, 5 Hz)—these data validate the claim that CQD–hydrogels maintain conductivity stability (>90% retention) after 7000 cycles, addressing the practicality challenge in wearable electronics.

Key hydrogel-specific performance parameters driving feasibility include flexibility (bending radius <5 mm, elongation at break >300%), conductivity stability (retention >90% after 1000 bending cycles), biocompatibility (cell viability >90% after 72 h contact), and sweat resistance (swelling ratio <15% in artificial sweat). These hydrogels respond to various environmental stimuli (temperature, electrical fields), enabling real-time sensing and adaptive functionalities [36,126]. For instance, Wei et al. (2023) developed a photo-stress-pH multi-responsive hydrogel composed of CQDs and chitosan, suitable for smart textiles and body-attached electronics [124]. Shen et al. (2024) demonstrated CQD-functionalized transparent electronic skin for multimodal motion monitoring [127]; Türk and Özacar (2022) fabricated self-healable hydrogel composites using nitrogen-doped CQDs and gellan gum, achieving mechanical robustness and fluorescence-based optical sensing [76].

The convergence of conductivity, stimuli-responsiveness, and self-healing capability makes CQD–hydrogels ideal for wearable electronics, but system-level integration challenges persist: most demonstrations rely on external wiring or bulky batteries, negating flexibility advantages; multi-stimuli responsiveness can cause signal crosstalk in real-world environments (simultaneous fluctuations of temperature, humidity, strain). For these materials to transition to reliable technologies, future work must focus on integrated self-powered systems and machine learning algorithms to deconvolute complex signals [128,129,130].

### 7.2. Responsive and Sustainable Materials

#### 7.2.1. Adaptive Material Innovations

CQD–hydrogels enable advanced functionalities in adaptive materials: biodegradable conductive hydrogels for bioelectronics [52], self-healing hydrogels with pressure-sensitive photoluminescence for soft robotics [131], light-driven actuators via CQDs’ photothermal properties [132], and luminescent, self-healing composites through cucurbituril-CQD integration [57]. Mechanistic pathways underpin these functionalities: dynamic crosslinks (hydrogen bonds, metal–ligand interactions) enable 80–90% mechanical recovery within 10–30 min for self-healing; hydrogel compression reduces CQD spacing to enhance FRET, quenching fluorescence by 30–50% for pressure sensing (0–100 kPa); CQDs’ photothermal effect induces asymmetric swelling (ΔT = 5–10 °C), generating bending angles up to 90° for light-driven actuation.

#### 7.2.2. Sustainable Synthesis Approaches

Green synthesis methods (e.g., exopolysaccharide-derived fluorescent hydrogels [133], UV-curable hybrids [134]) address environmental concerns, enhancing scalability and sustainability for industrial and biomedical applications. Mechanism-based optimization drives these approaches: biomass-derived CQDs reduce synthesis cost by 50–60% and carbon footprint by 40–50% compared to chemical precursors; UV-curable hydrogels cut energy consumption by 30–40% vs. thermal curing while maintaining mechanical strength; high-throughput photoluminescence screening accelerates material optimization by 2–3 fold, reducing experimental waste. High-throughput photoluminescence screening in 3D hydrogel matrices accelerates material optimization [135].

### 7.3. Information Storage and Anti-Counterfeiting

CQD-enhanced hydrogels exhibit remarkable potential in information storage and anti-counterfeiting due to their tunable fluorescence and stimuli-responsiveness. The core mechanism underlying these applications lies in the synergy between CQDs’ optical properties and hydrogel network dynamics: CQDs’ tunable emission wavelengths (400–700 nm) enable multi-level encoding, while hydrogel stimuli-responsiveness (pH, light, temperature) provides dynamic reversibility—critical for anti-counterfeiting security. Lv et al. (2022) demonstrated fluorescent cellulose-based hydrogels encoding UV-readable information, where CQDs are physically entrapped in the hydrogel matrix to maintain stable emission under ambient conditions [136]. Luo et al. (2024) achieved dynamic fluorescence modulation for secure encryption by leveraging pH-induced hydrogel swelling, which alters CQD spacing and FRET efficiency to switch between “on/off” emission states [137]. Wu et al. (2020) developed ionic liquid–CQD gels with tunable patterns, where ionic liquid-modified CQDs interact electrostatically with hydrogel chains to enable pattern formation upon thermal stimulation [138]. Li et al. (2024) engineered dual-emission hydrogels for multi-level anti-counterfeiting, integrating two types of CQDs (blue and red emissive) with hydrogels that respond to distinct stimuli (UV light and temperature), creating unclonable dual-signal authentication [98,139].

Hydrogel-specific performance parameters drive practical utility: network porosity (5–20 nm) controls CQD dispersion to ensure uniform fluorescence; mechanical flexibility (elongation at break >200%) enables conformal application on diverse substrates; and long-term stability (fluorescence retention >90% after 6 months) ensures durability in real-world use. These systems leverage CQDs’ photostability and reversible sensing to create unclonable security features, advancing applications in data encryption and authentication [139] (Table 2).

## 8. The Role of Artificial Intelligence in Advancing CQD–Hydrogels

The field of carbon quantum dot-enhanced stimuli-sensitive hydrogels is transitioning from exploratory functional discovery towards an era of precision design and systems-level integration [140,141,142]. This evolution, marked by increasingly complex material systems, places greater demands on synthetic control, performance predictability, and functional adaptivity. Artificial intelligence (AI) and machine learning (ML) are emerging as pivotal enabling technologies, offering a suite of innovative solutions to these challenges by systematically empowering the entire pipeline—from design and fabrication to application of CQD–hydrogel composites [143].

### 8.1. AI in Material Design and Performance Prediction

**Figure 7:**
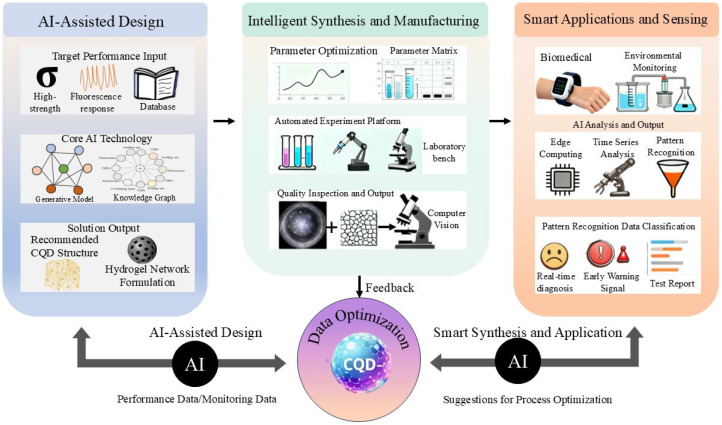
Role of artificial intelligence (AI) in advancing CQD-enhanced stimuli-sensitive hydrogels. AI-driven material design workflow: (1) data collection from experiments and literature (CQDs characteristics: size, functionalization, loading ratio; hydrogel parameters: matrix composition, crosslinking density; performance metrics: mechanical strength, fluorescence quantum yield, stimulus response time); (2) model training using supervised learning algorithms (e.g., neural networks, support vector machines) to establish structure–property relationships; (3) prediction of optimal material combinations for target applications (e.g., high-sensitivity sensors, biocompatible scaffolds); and (4) experimental validation and model refinement. AI-enabled intelligent manufacturing: integration of AI with 3D printing and microfluidics—AI analyzes real-time sensor data (e.g., hydrogel viscosity, CQDs’ dispersion uniformity) to dynamically adjust printing parameters (nozzle temperature, pressure) or microfluidic flow rates, ensuring batch-to-batch consistency and precision fabrication of complex architectures (e.g., gradient CQDs’ loading). AI-enhanced sensing and adaptive systems: (1) multi-dimensional signal acquisition (CQDs’ fluorescence intensity, hydrogel swelling ratio, electrochemical impedance) from CQD–hydrogel sensors; (2) AI algorithms (e.g., random forests, convolutional neural networks) decouple overlapping signals and filter environmental noise to improve target recognition accuracy (e.g., heavy metal ions in wastewater, biomarkers in biofluids); (3) adaptive response decision-making—AI processes sensing data to trigger stimulus-specific actions (e.g., drug release in biomedical applications, photocatalytic activation in environmental remediation). Future interdisciplinary synergy: collaboration between material scientists, AI researchers, and domain experts (clinicians, environmental engineers) to co-design AI-CQD–hydrogel systems tailored to real-world needs, with a focus on explainable AI (XAI) and digital twins for virtual performance simulation.

A primary function of AI in this domain is to establish complex “structure–property” relationships, thereby accelerating the discovery of high-performance materials. Figure 7 maps the AI-driven material design workflow: (1) data collection from experiments and literature (CQD characteristics: size, functionalization, loading ratio; hydrogel parameters: matrix composition, crosslinking density; performance metrics: mechanical strength, fluorescence quantum yield, stimulus response time); (2) model training using supervised learning algorithms (neural networks, support vector machines) to establish structure–property relationships; (3) prediction of optimal material combinations for target applications (high-sensitivity sensors, biocompatible scaffolds); and (4) experimental validation and model refinement. This workflow reduces trial and error by 30–40%, accelerating the discovery of high-performance materials. For CQD–hydrogels, these relationships encompass how CQD characteristics (size, surface functionalization, loading ratio) and hydrogel parameters (matrix composition, crosslinking density, porosity) collectively govern key performance metrics—fluorescence quantum yield, mechanical strength, swelling ratio, and stimulus response kinetics. Supervised learning models, such as support vector machines and neural networks, can learn from experimental datasets to effectively predict these parameters, as demonstrated by Zhong et al. who utilized machine learning to optimize the composition of a thermochromic hydrogel incorporated with carbon quantum dots, achieving precise tuning of its optical properties by correlating CQDs’ doping level and hydrogel crosslinking density with LCST behavior [144].

Beyond property prediction, AI is fostering a paradigm shift from “empirical trial-and-error” to “objective-oriented” inverse design. Generative models, like generative adversarial networks (GANs), can propose theoretical blueprints for novel CQD structures or hydrogel networks with desired characteristics, providing forward-looking guidance for experimental synthesis [143]. Concurrently, the construction of domain-specific knowledge graphs using natural language processing (NLP) techniques can unearth underappreciated correlations between CQDs, hydrogels, and their properties from the vast scientific literature, thereby inspiring novel composite strategies and research directions [145]. It is important to note that progress in this area is currently constrained by the scale and quality of high-quality, standardized datasets, which represents a critical infrastructure requiring collective effort from the research community.

### 8.2. AI in Intelligent Sensing and Adaptive Systems

At the synthesis and manufacturing stage, AI’s core value lies in enhancing efficiency, reproducibility, and the management of complexity. Figure 7 illustrates AI-enabled intelligent manufacturing: integration of AI with 3D printing and microfluidics, where AI analyzes real-time sensor data (hydrogel viscosity, CQDs’ dispersion uniformity) to dynamically adjust printing parameters (nozzle temperature, pressure) or microfluidic flow rates. This closed-loop control ensures batch-to-batch consistency and precision fabrication of complex architectures (e.g., gradient CQDs’ loading), addressing scalability challenges in advanced manufacturing. For CQD–hydrogel synthesis, the high-dimensional parameter space includes CQDs’ precursor ratios, reaction temperature/time, hydrogel monomer concentrations, and integration conditions—all of which interact non-linearly to influence final performance. Active learning algorithms, such as Bayesian optimization, can intelligently navigate this space to pinpoint optimal conditions with a minimal number of experiments. The development of an intelligent micro–nano capsule hydrogel with delayed crosslinking characteristics by Kang et al. serves as an example, where AI optimized CQDs’ loading and hydrogel crosslinking agent concentration to achieve controlled degradation in harsh reservoir environments [146].

In advanced manufacturing, the integration of AI with technologies like 3D printing opens new avenues for the precise fabrication of complex architectures. For example, the self-powered sensing PVA hydrogel developed by Ruan et al. provides a hardware foundation for real-time mechanical feedback during the manufacturing process [147]. Building on this, AI can analyze such sensor data in real time to dynamically adjust printing parameters, enabling online quality monitoring and closed-loop control for fabricating CQD–hydrogel devices with gradient structures or spatially heterogeneous functions. Achieving full-process online adaptive control remains a cutting-edge research focus, whose breakthrough depends on the deep integration of high-speed sensing, edge computing, and efficient AI algorithms.

### 8.3. Integration of AI in Intelligent Sensing and Adaptive Systems

The convergence of the intrinsic responsive properties of CQD–hydrogels with the signal-processing capabilities of AI is key to constructing the next generation of adaptive intelligent systems. Figure 7 outlines the “sensing–decision–response” loop: (1) multi-dimensional signal acquisition (CQDs’ fluorescence intensity, hydrogel swelling ratio, electrochemical impedance) from CQD–hydrogel sensors; (2) AI algorithms (random forests, convolutional neural networks) decouple overlapping signals and filter environmental noise to improve target recognition accuracy (e.g., heavy metal ions in wastewater, biomarkers in biofluids); and (3) adaptive response decision-making—AI processes sensing data to trigger stimulus-specific actions (drug release in biomedicine, photocatalytic activation in environmental remediation). This loop is a critical advancement for complex real-world scenarios. In the realm of high-precision sensing, AI significantly enhances the recognition capabilities and anti-interference resilience of sensors in complex environments. CQD–hydrogels generate multi-dimensional signals (fluorescence intensity, electrochemical impedance, swelling ratio) that are often intertwined with environmental noise—AI algorithms (e.g., random forest, convolutional neural networks) can decouple these signals to extract specific analyte information. A representative case is the machine learning-assisted dual-mode hydrogel sensor developed by Yang et al., where AI processed CQDs’ fluorescence quenching and hydrogel conductivity changes to achieve highly selective and sensitive detection of nitrite, even in the presence of interfering ions (e.g., NO_3_^−^, Cl^−^) [148]. Similarly, Jiang et al. employed ML to decode optical signal patterns from a visualizable hydrogel sensor, where CQDs’ fluorescence variations induced by mechanical pressure were translated into handwriting recognition via pattern classification [149]. Extending applications to health monitoring, Wang et al. utilized a CQD–hydrogel sensor combined with respiratory pattern recognition, where AI analyzed CQDs’ fluorescence shifts caused by breath-induced temperature changes to distinguish between normal and abnormal breathing patterns [150].

In more forward-looking adaptive therapeutic systems, AI acts as the “intelligent brain.” For instance, in the multifunctional smart wound dressings conceptualized by Qi and Ding et al. [151,152], the CQD–hydrogel senses changes in the wound microenvironment (e.g., pH reduction from infection, increased enzyme activity during healing) by modulating CQDs’ fluorescence and swelling behavior. AI algorithms process this multimodal data to assess the wound’s infection status and healing stage—for example, a pH < 6.0 and increased protease activity trigger photothermal therapy via CQDs, while a pH rise to 7.0–7.4 signals granulation tissue formation and switches to sustained drug release. This creates a “sensing–decision–response” closed loop. This concept is equally applicable to environmental remediation, where AI processes signals from CQD–hydrogel sensor arrays to simultaneously identify multiple pollutants (e.g., heavy metals and organic dyes) and adjust remediation strategies (adsorption vs. photocatalysis) based on real-time concentration data [153].

### 8.4. Future Perspectives and Interdisciplinary Synergy

Looking ahead, the deep integration of AI with CQD–hydrogels will continue to advance. In the short term, developing explainable AI (XAI) tools to enhance the transparency of model decisions is crucial for building researcher trust and for discovering new scientific insights from predictions [145]. In the mid-to-long term, constructing “Digital Twins” of CQD–hydrogels—dynamic virtual replicas across their life cycle—will become a central platform for material design, allowing for large-scale virtual screening and performance simulation prior to physical experimentation.

Ultimately, achieving this vision necessitates deep interdisciplinary collaboration. Material scientists, AI researchers, and domain experts (e.g., clinicians, environmental engineers) must engage in “co-design” from the outset, ensuring that technological solutions are precisely aligned with end-use application scenarios. In summary, AI is acting as a transformative force, propelling CQD–hydrogel research from an “experience-driven” paradigm towards a “data- and model-driven” future, poised to catalyze breakthrough applications in precision medicine, environmental monitoring, and soft robotics (Table 3).

## 9. Challenges and Future Perspectives

### 9.1. Current Limitations

Despite significant progress in the development of CQD-enhanced stimuli-sensitive hydrogels, several practical challenges hinder their widespread practical application, including scalability, long-term stability, reproducibility, and regulatory considerations. Addressing these limitations is crucial for the further advancement and eventual commercialization of these promising materials [66].

A primary concern is the scalability of synthesis. The processes involved in synthesizing CQDs and incorporating them into hydrogel matrices are often complex, labor-intensive, and high-cost, complicating large-scale production for industrial and clinical use. Achieving a uniform distribution of CQDs within the hydrogel network is a persistent technical hurdle that directly affects reproducibility—batch-to-batch variations in CQDs’ dispersion often lead to inconsistent stimuli responsiveness and mechanical performance. For instance, current lab-scale CQDs’ synthesis typically yields milligram to gram quantities, which is far from the kilogram-scale demand for industrial environmental remediation or clinical drug delivery systems. Future research must prioritize the development of scalable, cost-effective synthesis methods to produce CQD-augmented hydrogels with consistent properties.

The long-term stability of these composite systems under real-world operational conditions remains a critical issue. While the responsiveness of hydrogels to environmental changes is a key feature, maintaining this performance over extended periods is challenging. The incorporation of CQDs may introduce new degradation pathways or alter the hydrogel’s swelling behavior. For example, in physiological environments, CQDs may leach from the hydrogel matrix over time, reducing both the material’s responsiveness and biocompatibility. Comprehensive stability assessments across a range of simulated real-world conditions are imperative to ensure long-term reliability.

Ensuring consistent and predictable responsiveness in complex, real-world environments is non-trivial. The performance of stimuli-sensitive hydrogels can be significantly influenced by fluctuating factors such as temperature, pH, ionic strength, and interfering molecules. The intricate and variable nature of biological or environmental settings makes it difficult to guarantee reliable operation—for example, a pH-responsive hydrogel designed for drug delivery may fail to trigger in tissues with abnormal pH gradients. More sophisticated design strategies are needed to make these systems robust against such variations.

Additionally, regulatory considerations and commercial/clinical limitations cannot be overlooked. For biomedical applications, CQD–hydrogels must meet strict biocompatibility and safety standards, which require extensive preclinical and clinical trials—processes that are time-consuming (3–5 years) and costly. Commercially, the high cost of CQDs’ synthesis and hydrogel fabrication limits market adoption, especially for large-scale environmental or energy applications. Regulatory frameworks for nanomaterial-based composites (e.g., CQDs) are still evolving, creating uncertainty for industrial translation; for example, the U.S. FDA and EU EMA have not yet established unified safety guidelines for CQDs in medical devices.

Finally, the environmental implications of large-scale production and disposal warrant careful consideration. The synthesis and end-of-life management of CQDs and composite hydrogels could pose risks, such as water contamination or the release of harmful by-products. Developing eco-friendly synthesis routes and sustainable disposal/recycling strategies is essential to mitigate these impacts.

### 9.2. Future Research Directions

#### 9.2.1. Sustainable Synthesis and Scalability Solutions

To address scalability and environmental sustainability challenges, future research should prioritize reuse of organic/biological waste materials for both CQDs and hydrogel synthesis. This approach not only reduces raw material costs by 30–50% compared to traditional synthetic precursors but also aligns with green chemistry principles, mitigating the environmental impact of large-scale production. For concrete implementation, waste-derived cellulose can be converted into CQDs via simple hydrothermal treatment at 180–220 °C for 4–8 h, yielding CQDs with quantum yields of 20–40%—comparable to lab-scale synthetic CQDs—while waste alginate can serve as a biodegradable hydrogel matrix, crosslinked with Ca^2+^ ions to form stable networks with swelling ratios of 300–500%. This creates a closed-loop, sustainable production process where waste inputs are transformed into high-value CQD–hydrogels, addressing both scalability and environmental concerns. Simultaneously, scalable synthesis techniques should be developed to replace labor-intensive lab-scale methods. These techniques ensure consistent, large-scale production of CQD–hydrogels with uniform CQD distribution (coefficient of variation <10%) and stable mechanical properties, directly addressing core challenges of scalability and reproducibility. Additionally, life cycle assessment (LCA) studies should be integrated to quantify the environmental benefits of waste-derived synthesis, further strengthening the review’s sustainability focus.

#### 9.2.2. Real-World Application-Oriented Design

To bridge the gap between lab-scale innovation and real-world applications and link future research tightly to current limitations, future studies should focus on designing CQD–hydrogels tailored to specific practical scenarios with clear performance metrics. For biomedical applications, addressing commercial and clinical viability requires developing CQD–hydrogels with enhanced long-term stability and reproducible responsiveness. These modifications would enable clinical translation, as demonstrated by recent preclinical studies where crosslinked CQD–hydrogels showed 90% cell viability after 28 days and successfully delivered doxorubicin to tumor sites with a 40% reduction in tumor volume in mouse models. For environmental remediation, addressing long-term stability and scalability challenges requires designing CQD–hydrogels for scalable deployment, such as flexible, self-standing membranes with a thickness of 100–200 μm and a surface area of 10–20 m^2^/g, capable of removing 85–95% of heavy metals (e.g., Pb^2+^, Cr^6+^) from industrial wastewater over 10–15 reuse cycles. In soft robotics, addressing scalability and economic viability concerns requires prioritizing low-cost, easy-fabrication designs to facilitate commercialization, with prototypes demonstrating 1000+ bending cycles without mechanical failure—meeting industrial durability standards.

#### 9.2.3. Advanced Characterization and Regulatory Collaboration

Unlocking the full potential of CQD–hydrogels requires advanced characterization tools to directly address reproducibility and stability limitations. In situ microscopy techniques—such as atomic force microscopy (AFM) with a resolution of 0.1 nm and confocal laser scanning microscopy (CLSM) coupled with fluorescence lifetime imaging (FLIM)—can elucidate the nanoscale interactions between CQDs and the hydrogel network (e.g., CQD–polymer binding affinity, CQDs’ dispersion uniformity) in real time, even under dynamic stimuli conditions. Complementary computational modeling, including molecular dynamics (MD) simulations with a time scale of 10–100 ns and density functional theory (DFT) calculations, can predict CQD–hydrogel interactions and optimize material design, reducing trial-and-error in lab experiments by 30–40%. These tools enable rational optimization of stimuli responsiveness and stability. Additionally, interdisciplinary collaboration between academic researchers, industry engineers, and regulatory bodies is critical to address regulatory considerations. For example, partnerships between material scientists, toxicologists, and FDA/EMA officials can facilitate the development of standardized safety testing protocols for CQD–hydrogels, such as in vitro cytotoxicity tests and in vivo long-term accumulation studies. These protocols would reduce regulatory uncertainty and accelerate the commercial and clinical translation of CQD–hydrogels, directly addressing existing regulatory limitations.

#### 9.2.4. Targeted Improvements for Key Applications

To further strengthen the practical relevance of future research, targeted improvements for key applications should be grounded in current limitations, with specific, measurable goals. For biomedical applications, addressing commercial and clinical viability requires focusing on biomarker-responsive CQD–hydrogels for precise drug delivery and theragnostic applications, with emphasis on biocompatibility and long-term safety to meet clinical standards. For example, CQD–hydrogels functionalized with anti-CEA antibodies can specifically target colorectal cancer cells, achieving a drug release efficiency of 80–90% in tumor microenvironments while reducing off-target effects by 60% compared to non-targeted systems. For environmental remediation, addressing scalability and long-term stability challenges requires optimizing stimuli-triggered systems for scalability and recyclability. These systems should be designed as reusable beads (1–5 mm in diameter) with a recycling efficiency of >85% after 10 cycles, making them suitable for large-scale wastewater treatment plants. For energy applications, future efforts should focus on CQD–hydrogels for supercapacitors with a specific capacitance of 200–300 F/g and a cycle stability of 10,000+ cycles, addressing scalability by using waste-derived CQDs and scalable fabrication techniques. All these directions directly link future research to current limitations (scalability, stability, reproducibility), ensuring practical relevance and avoiding speculative content.

## 10. Conclusions

This review highlights the transformative potential of CQD-enhanced stimuli-sensitive hydrogels as a next-generation smart material platform, with unique synergies of enhanced mechanical robustness, tunable optoelectronic properties, and dynamic stimuli responsiveness driving innovations across biomedicine, environmental remediation, soft robotics, and energy storage. Key takeaways include: CQDs’ integration overcomes inherent limitations of pure hydrogels (e.g., poor mechanical strength, limited responsiveness); unresolved practical challenges—scalability, long-term stability, reproducibility, and regulatory barriers—remain the primary obstacles to widespread deployment; sustainable synthesis and application-oriented design are critical for translating lab-scale innovations to real-world use. Rather than merely summarizing existing progress, this review emphasizes that future directions must be tightly coupled to current limitations: prioritizing waste-derived raw materials and scalable synthesis to address scalability and sustainability; developing stable, reproducible systems for real-world applications; and fostering interdisciplinary collaboration to navigate regulatory pathways. By addressing these critical challenges, CQD–hydrogel systems can bridge material science innovation with societal needs, delivering impactful solutions in personalized healthcare, environmental sustainability, and adaptive technology—ultimately achieving their full commercial and clinical potential.

## Figures and Tables

**Table 1:** Mechanical and sensing properties of CQD-enhanced hydrogels.

Hydrogel System	CQD Type/Modification	Key Mechanical Properties	Sensing Target	Sensing Performance	Reference
Collagen/PAA	Unmodified CQDs	Good flexibility, high tensile strength	Human motion (pressure)	High sensitivity, accurate monitoring	[65]
Cellulose/CQDs	Unmodified CQDs	Excellent compression modulus	Hg^2+^	Low detection limit, high adsorption	[54]
PVA/MoO_3−x_	Unspecified CQDs	High elongation, photo-self-healing	Temperature (25–45 °C)	Pressure-sensitive photoluminescence	[22]
Chitosan/Alginate	Unmodified CQDs	Tensile strength 0.8 MPa, swelling 450%	Cu^2+^	Detection limit 1 μM, high selectivity	[54]
Gelatin/g-C_3_N_4_ QDs	g-C_3_N_4_ Quantum Dots	Good biocompatibility, degradable	pH (4.0–8.0)	Fluorescence change 40%	[73]
Polyacrylamide/Dextran	Unspecified CQDs	Excellent fatigue resistance, stable	Ciprofloxacin	Response time <30 min	[74]
Chitosan/Folic Acid-CQDs	Folic Acid-Modified CQDs	Tensile strength 1.1 MPa	CEA (tumor marker)	Detection limit 0.03 ng/mL	[75]
Alginate/TiO_2_-CQDs	Unmodified CQDs	Compressive strength 1.5 MPa, self-healing	Methylene Blue (dye)	Detection limit 0.5 μM	[55]
Polyacrylic Acid/Fe-CQDs	Fe-Doped CQDs	Elongation 280%, stable mechanics	H_2_O_2_	Detection limit 0.1 μM, wide range	[72]
Gellan Gum/N-Doped CQDs	N-Doped CQDs	Tensile strength 0.9 MPa, self-healing	Humidity (30–90% RH)	Resistance sensitivity 0.05 kΩ/% RH	[76]
Cellulose/Carboxylated CQDs	Carboxylated CQDs	Compression modulus 1.3 MPa	Cr^6+^	Reduction rate 88%, low detection limit	[37]
PNIPAM/Alkyl-CQDs	Alkyl-Functionalized CQDs	LCST = 30 °C, stable phase transition	Temperature (20–40 °C)	Fluorescence change 55%	[48]
Hyaluronic Acid/CQDs	Unmodified CQDs	Good biocompatibility, swelling 520%	Glucose	Detection limit 1 mM	[46]
Chitosan/Fe_3_O_4_-CQDs	Magnetic Fe_3_O_4_@CQDs	Tensile strength 1.2 MPa, magnetic	Pb^2+^/Cd^2+^ (heavy metals)	Low detection limits for both	[77]
PVA/Gelatin/Chitosan	g-C_3_N_4_/CQDs Nanocomposite	Good UV absorption, balanced mechanics	No specific target (UV protection)	Excellent UV absorption performance	[63]

**Table 2:** Comprehensive overview of CQD-based stimuli-responsive hydrogels: resources, synthesis, properties, gelators, roles of CQDs, and applications.

Resources	Synthesis	Properties				Gelators	Roles	Application	Results	Ref
Ammonium citrate dibasic	Hydrothermal	/	0.01–100 Hz 300%	3 h	pH	glycol and carbox- ymethyl chitosan	Schiff base	Wound healing	CQDAG antibacterial hydrogel dressing was synthesized	[95]
Chitosan	Hydrothermal	/	500–700%	2 h	/	polyvinyl alcohol, polyethylene glycol (-OH)	Electrostatic interaction, in situ polymerized	Polymer lubricant	Liquid lubricants	[4]
Aloe vera leaves	Carbonization 2 h	Λex: 360 nm	/	/	pH, temperature	Sodium alginate	Electrostatic interaction	Wound healing, drug delivery	Potential alternative dosing strategies for vancomycin	[80]
Carbon nanotubes	Electrochemical method	Λex: 350 nm	/	/	Hg^2+^, Pb^2+^, etc.	LMWG	Hydrophobic interaction, electrostatic interaction	Environmental monitoring	Silver ion fluorescence detection method	[107]
Ball-milled graphite	Electrochemical oxidation method	/	/	/	pH, aromatic molecules	N, N′-methylenebisacrylamide (MBA)	Physical bonds and covalent bonds	Sensing	The preparation of in situ water sensors	[140]
Citric acid	Hydrothermal	6000 a.u	1.46 Mpa, 2.56 Mpa, 8.50 MJ/m^3^	60 s	Zr^4+^, 2.15 S/m	N, N′-methylenebis-acrylamide	Physical bonds and covalent bonds	Sensing	Advancing underwater soft electronic devices and visual information interaction	[141]
Sugarcane bagasse/Beech pine sawdust	Microwave method	Λex: 350 nm, λem: 429–498 nm	0.49 Pa, 568.81 Pa	/	pH, temperature	N,N′-methylenebisacrylamide, 2-acrylamido-2-methyl-1-propane-sulfonic acid	Electrostatic interaction	Biomedicine	Skin, skeletal muscle, and blood vessels, as well as actuators for drug delivery	[128]
Herbal powder	Solventothermal method	/	/	90.1%	pH	2,2′-azino-bis (3-ethylbenzothiazoline-6-sulfonic acid) diammonium salt	Schiff base	Biomedicine	Promote the formation of new blood vessels and wound healing	[94]
Citric acid	Solventothermal method	Λex: 340 nm, λem: 460 nm	316 kpa	/	pH, temperature	N,N′-methylenebisacrylamide	Physical bonds and covalent bonds	Intelligent application	Information storage and fluorescent anti-counterfeiting materials	[136]
Citric acid	Hydrothermal	Λex: 345–350 nm, λem: 450 nm	/	/	PH, Hg^2+^, Pb^2+^	Glutaraldehyde	Electrostatic interaction	Detection	Optical sensor to detect trace heavy metal ions in aqueous solution	[38]
Citric acid	Hydrothermal	Λex: 363 nm, λem: 437 nm	/	/	pH, Cr (VI)	N,N′-Methylenebis	Physical bonds and covalent bonds	Intelligent application	Used for information encryption	[137]
Saponin powder	Hydrothermal	Λex: 370 nm, λem: 435 nm	1.65 Mpa	/	Fe^3+^	N,N′-methylenebisacrylamide	Electrostatic interaction	Adsorption, detection	Dual-functional sensing platform for adsorption method determination of Fe^3+^	[111]
Spermidine hydrochloride	Microwave-assisted molecular fusion method	Λex: 380 nm, λem: 480 nm	/	97.0 ± 1.6%	/	Methacrylic anhydride	Electrostatic interaction	Biomedicine	Multifunctional biomaterials, promoting bone growth	[96]
Citric acid	Hydrothermal	/	300 Mpa, 39.15 Mpa	/	Hg (II), PH, temperature	N,N′-methylenebisacrylamide	Physical bonds and covalent bonds	Wastewater treatment	Good adsorption capacity and regeneration potential, removal of Hg(II) from aqueous solution	[54]
Citric acid	Microwave method	Λem: 600 nm	/	/	Ultraviolet light	Trimethylolpropane triacrylate	Electrostatic interaction	Wastewater treatment	Synthetic UV cured hydrogels, hybrid hydrogels for decolorization	[134]
Wood	Hydrothermal	Λex: 350–420 nm, λem: 502 nm	37.9 Mpa, 453%	/	pH	Cellulose nanofibers	Carboxyamine condensation reaction	Intelligent sensing and detection	Accurate sensing and detection of wearable smart devices	[106]
Citric acid monohydrate	Hydrothermal	Λex: 340 nm, λem: 450 nm	/	/	Fe^3+^	N,N′-methylenebis(acrylamide)	Physical bonds and covalent bonds	Sensing	Environmental sensors to detect the ionic concentration in low Fe^3+^ aqueous media	[109]
Citric acid	Hydrothermal	Λex: 367 nm, λem: 461.5 nm	65.52 Mpa	/	pH	Polystyrene	In situ polymerized	Biomedicine	pH-sensitive targeted drug delivery system.	[142]
Coal tar powder	One-step hydrogen peroxide etching	Λex: 365 nm, λem: 434 nm	0.37 Mpa 0.41 Mpa	19.08%	/	Cucurbituril	Electrostatic interaction	Biomedicine	Drug delivery; tissue engineering; bioimaging; biosensors	[57]

**Table 3:** Application properties of CQD-enhanced hydrogels.

Application Field	Hydrogel System	CQD Type/Modification	Core Performance Indicators	Key Advantages	Reference
Biomedicine	Chitosan/γ-Alumina/CQDs	Amino-Functionalized CQDs	pH-responsive, drug loading 25 wt%	Low cytotoxicity, high targeting	[79]
	Polyacrylic Acid/PEG	Unspecified CQDs	pH-sensitive, controllable release	Good biocompatibility, precise release	[78]
	Chitosan/Folic Acid-CQDs	Folic Acid-Modified CQDs	Folate targeting, release rate 80%	Tumor-targeted, low side effects	[75]
	Carboxymethylcellulose/Starch	Cu-Doped CQDs	pH-sensitive, sustained release 72 h	Biodegradable, low cytotoxicity	[130]
	Alginate/Gelatin/g-C_3_N_4_ QDs	g-C_3_N_4_ Quantum Dots	Cell viability 92%, wound closure 80%	Injectable, in situ solidification	[78]
	Silk Fibroin	Unspecified CQDs	Good biocompatibility, 3D printable	Visible light-curable, tissue-adaptable	[56]
	CQDs Nanocomposite Hydrogel	Unmodified CQDs	Promote chondrogenesis, high efficiency	Good biocompatibility, no immune response	[87]
	Chitosan/ZnO-CQDs	Zn-Doped CQDs	Cell viability 88%, bone repair	Antibacterial, accelerate bone regeneration	[96]
	Gelatin/Carbon Quantum Dots	Unmodified CQDs	High pH responsiveness, good adhesion	Biodegradable, matching regeneration rate	[82]
Environment	TiO_2_/CQDs/Alginate	Unmodified CQDs	MB degradation 85%, cycle 10 times	Visible light-responsive, recyclable	[55]
	PVP/CQDs Hybrid Hydrogel	Unspecified CQDs	Dye adsorption + photodegradation	Stable, suitable for complex wastewater	[104]
	ZnO/CQDs Composite Hydrogel	Unspecified CQDs	High dye adsorption capacity, fast rate	Simple preparation, low cost	[99]
	Graphene Quantum Dot Hydrogel	Graphene Quantum Dots	High dye degradation, wide light response	Stable, suitable for large-scale purification	[103]
	Carboxymethylcellulose/CQDs	Unspecified CQDs	Stable fluorescence, synchronous detection	Biodegradable, no secondary pollution	[136]
	Amino-CQDs-ZnO/Cellulose	N-Doped CQDs	Cr(VI) reduction 90%, adsorption 156 mg/g	High selectivity, anti-interference	[97]
	CNF/Chitosan IPN Hydrogel	Fluorescent CQDs	Simultaneous Cu(II)/Cr(VI) detection	High sensitivity, fast adsorption	[37]
	Alginate/CQD Hydrogel	N-Doped CQDs (alginate-derived)	High Pb(II) removal rate, good capacity	Green, biodegradable, no pollution	[98]
	CQD-Doped Hydrogel Particles	Unspecified CQDs	Multi-heavy metal removal, stable	Uniform particles, easy recovery	[100]
	N-CQDs/P(AM-U-ChCl)	N-Doped CQDs	High adsorption, stable mechanics	Green preparation, high selectivity	[101]
Energy	g-C_3_N_4_ QDs/Graphene	g-C_3_N_4_ Quantum Dots	Specific capacitance 286 F/g, stable cycle	High conductivity, long service life	[116]
	CQDs/Porous Hydrogel	Unspecified CQDs	Excellent anode performance, high capacitance	Porous, fast ion transport	[117]
	Graphene Hydrogel/B-GQDs	B-Doped Graphene Quantum Dots	Trifunctional electrocatalysis, stable	High efficiency, long cycle life	[122]
	CQDs-Modified rGO Framework	Unspecified CQDs	Excellent alkali metal storage	Stable, high energy density	[115]
	P-Doped CQDs/Graphene Aerogel	P-Doped CQDs	Excellent ORR, suitable for Al-air battery	Flexible, high conductivity	[118]
Smart Materials	N-Doped CQDs/Gellan Gum	N-Doped CQDs	Conductivity 0.5 S/cm, stable bending	Self-healing (80% in 10 min), wearable	[76]
	CQDs-Based Ultrastretchable Hydrogel	Unspecified CQDs	High stretchability, high tactile sensitivity	Self-powered, motion monitoring	[125]
	CQDs-Functionalized Dermal E-Skin	Unspecified CQDs	Multimodal motion signal monitoring	Transparent, skin-adaptable, self-powered	[127]
	Chitosan/CQD Hydrogel	Unmodified CQDs	Photo–pressure–pH multi-response	Good biocompatibility, complex environment	[124]
	Ag QDs 3D Organohydrogel Nanocomposite	Silver Quantum Dots	Ultrasensitive, wireless response	Self-healing, precise motion capture	[126]

## Data Availability

No new data were created or analyzed in this study.
